# IκBζ regulates the development of nonalcoholic fatty liver disease through the attenuation of hepatic steatosis in mice

**DOI:** 10.1038/s41598-022-15840-0

**Published:** 2022-07-08

**Authors:** Hideki Ishikawa, Morisada Hayakawa, Nemekhbayar Baatartsogt, Nao Kakizawa, Hiromi Ohto-Ozaki, Takashi Maruyama, Kouichi Miura, Koichi Suzuki, Toshiki Rikiyama, Tsukasa Ohmori

**Affiliations:** 1 Department of Surgery, Saitama Medical Center, Jichi Medical University, 1-847 Amanuma, Omiya, Saitama 330-8503 Japan; 2 Department of Biochemistry, Jichi Medical University School of Medicine, 3311-1 Yakushiji, Shimotsuke, Tochigi 329-0498 Japan; 3 Center for Gene Therapy Research, Jichi Medical University, 3311-1 Yakushiji, Shimotsuke, Tochigi 329-0498 Japan; 4 Mucosal Immunology Section, National Institute for Dental and Craniofacial Research, National Institutes of Health, Bethesda, MD 20852 USA; 5 Division of Gastroenterology, Department of Medicine, Jichi Medical University School of Medicine, 3311-1 Yakushiji, Shimotsuke, Tochigi 329-0498 Japan

**Keywords:** NF-kappaB, Non-alcoholic fatty liver disease

## Abstract

IκBζ is a transcriptional regulator that augments inflammatory responses from the Toll-like receptor or interleukin signaling. These innate immune responses contribute to the progression of nonalcoholic fatty liver disease (NAFLD); however, the role of IκBζ in the pathogenesis of NAFLD remains elusive. We investigated whether IκBζ was involved in the progression of NAFLD in mice. We generated hepatocyte-specific IκBζ-deficient mice (*Alb-Cre; Nfkbiz*^*fl*/*fl*^) by crossing *Nfkbiz*^*fl*/*fl*^ mice with *Alb-Cre* transgenic mice. NAFLD was induced by feeding the mice a choline-deficient, L-amino acid-defined, high-fat diet (CDAHFD). CDAHFD-induced IκBζ expression in the liver was observed in *Nfkbiz*^*fl*/*fl*^ mice, but not in *Alb-Cre; Nfkbiz*^*fl*/*fl*^ mice. Contrary to our initial expectation, IκBζ deletion in hepatocytes accelerated the progression of NAFLD after CDAHFD treatment. Although the increased expression of inflammatory cytokines and apoptosis-related proteins by CDAHFD remained unchanged between *Nfkbiz*^*fl*/*fl*^ and *Alb-Cre; Nfkbiz*^*fl*/*fl*^ mice, early-stage steatosis of the liver was significantly augmented in *Alb-Cre; Nfkbiz*^*fl*/*fl*^ mice. Overexpression of IκBζ in hepatocytes via the adeno-associated virus vector attenuated liver steatosis caused by the CDAHFD in wild-type C57BL/6 mice. This preventive effect of IκBζ overexpression on steatosis was not observed without transcriptional activity. Microarray analysis revealed a correlation between IκBζ expression and the changes of factors related to triglyceride biosynthesis and lipoprotein uptake. Our data suggest that hepatic IκBζ attenuates the progression of NAFLD possibly through the regulation of the factors related to triglyceride metabolism.

## Introduction

Nonalcoholic fatty liver disease (NAFLD), which is a hepatic manifestation of metabolic syndrome or insulin resistance, has become a major healthcare issue worldwide^1–3^. The clinical range of NAFLD is broadly classified into two types^4–6^. The first is the simple nonalcoholic fatty liver (NAFL), in which lipids are deposited in hepatocytes; the second is nonalcoholic steatohepatitis (NASH), which involves the progression from NAFL to inflammation and fibrosis of the liver, and then to more severe manifestations of the disease, cirrhosis and hepatocellular carcinoma^2,7^. NASH is also associated with a high risk of acute complications and mortality, including cardiovascular events and extrahepatic malignancies^8–11^. Despite an increasing trend in NAFLD cases relative to the expansion of modern industrialized economies and the resulting global prevalence of obesity, there is no effective pharmacological therapy or cure to control the progression of NAFLD^12,13^. The elucidation of the pathogenesis and underlying mechanisms of NAFLD, therefore, remains an important challenge for the development of disease treatment and prevention.

Although the molecular pathogenesis of NASH has not been fully elucidated, several potential mechanisms have been indicated. The accumulation of triglycerides in hepatocytes results in an inflammatory response that is attributed to innate immunity, lipotoxicity, oxidative stress, endoplasmic reticulum stress, and autophagy, which are common mechanisms in the pathogenesis of NAFLD progression^14,15^. These subcellular responses activate intracellular pathways that lead to proinflammatory signals. Of these, NF-κB activation is a major intracellular event^16,17^. NF-κB regulates the expression of numerous proinflammatory proteins, including cytokines, chemokines, and adhesion molecules. NF-κB activation has been observed in several different animal models of NASH, as well as in humans, and is essential for the development of steatohepatitis^18,19^. The translocation of activated NF-κB into the nucleus has been demonstrated to activate the transcription of numerous proteins^20^.

IκBζ, which is encoded by the *Nfkbiz* gene, is a member of the nuclear IκB family, which acts as a transcriptional regulator of NF-κB signaling^21^. IκBζ is rapidly and robustly induced by NF-κB activation and amplifies the expression of secondary response genes, which enhance cytokine and chemokine production to regulate innate immune systems^21,22^. We have previously demonstrated that IκBζ augments interleukin (IL)-33-dependent cytokine and chemokine production in mast cells^23^. IκBζ also enhances Toll-like receptor (TLR), IL-1, IL-17, and IL-36 signaling^22,24,25^. Despite immune response augmentation, the disruption of IκBζ signaling increases cellular apoptosis in epithelial cells, which leads to the accumulation of lymphocytes and Sjögren syndrome-like autoimmune disease^26^. In addition, mutations in *NFKBIZ* are highly prevalent in the epithelium of ulcerative colitis patients and may prevent the progression of colorectal cancer^27^. Collectively, IκBζ is shown to have pleiotropic cellular functions, including immune response, apoptosis, and tumor progression. However, the specific role of IκBζ in the progression of NAFLD has not been determined. In this study, we have investigated the involvement of IκBζ in the progression of NAFLD in hepatocyte-specific *Nfkbiz*-deleted mice.

## Results

### Induction of IκBζ in the liver of mice during NAFLD development

**Figure 1:**
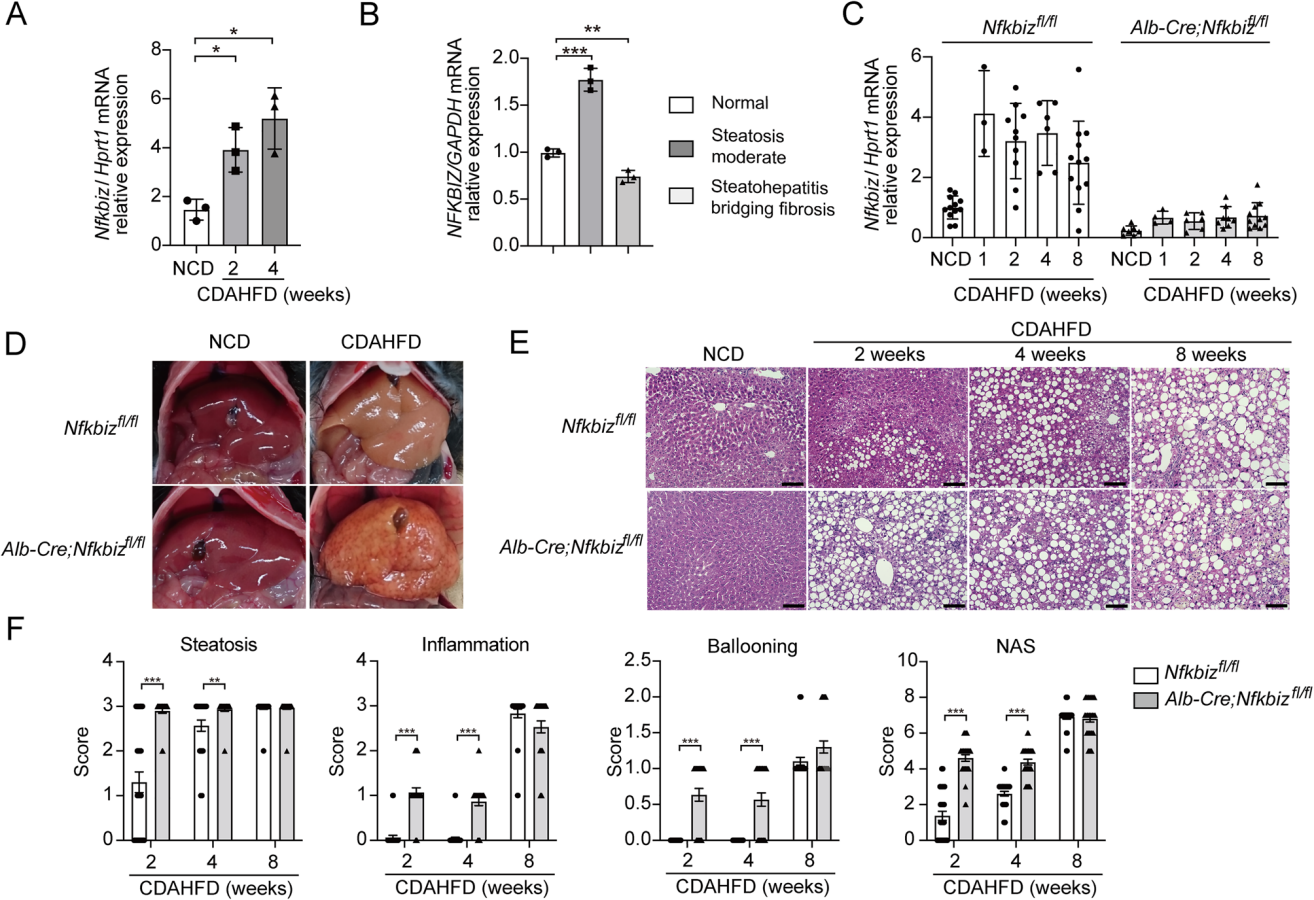
*Alb-Cre; Nfkbiz*^*fl/fl*^ mice accelerate the development of NAFLD. (**A**) C57BL/6 mice were fed an NCD or CDAHFD for 4 weeks. mRNA induction of *Nfkbiz* in the liver was assessed using real-time RT-PCR and was expressed as the fold increase in the *Nfkbiz*/*Hprt1* ratio. Values are expressed as the mean ± SD (n = 3). (**B**) mRNA expressions of *NFKBIZ* in the human normal liver, moderate steatosis, and steatohepatitis with bridging fibrosis were assessed using real-time RT-PCR. Values are expressed as the mean ± SD (triplicate experiment from one sample). (**C**) *Nfkbiz*^*fl/fl*^ and *Alb-Cre; Nfkbiz*^*fl/fl*^ mice were fed a CDAHFD or NCD for 8 weeks. mRNA induction of *Nfkbiz* in the liver was assessed using real-time RT-PCR. Values are expressed as the mean ± SD (n = 3–12 in each point). (**D**) Representative macroimages of the liver obtained from *Nfkbiz*^*fl/fl*^ and *Alb-Cre; Nfkbiz*^*fl/fl*^ mice fed an NCD or CDAHFD for 8 weeks. (**E**) Representative image of hematoxylin–eosin-stained liver sections obtained from *Nfkbiz*^*fl/fl*^ and *Alb-Cre; Nfkbiz*^*fl/fl*^ mice fed an NCD or CDAHFD for 8 weeks. Bar = 100 µm. (**F**) NAFLD activity score (NAS) was performed by a blinded researcher. The scores of hepatocellular steatosis (Steatosis), lobular inflammation (Inflammation), and ballooning (Ballooning), as well as total NAS calculated by summing up the scores (NAS), are expressed as the mean ± SD (10 independent fields from per mouse, three mice per experiment). *P < 0.05, **P < 0.01, ***P < 0.001. CDAHFD, choline-deficient, L-amino acid-defined, high-fat diet; NCD, normal chow diet.

To examine the role of IκBζ in the progression of NAFLD, we first examined the expression of IκBζ after a choline-deficient, L-amino acid-defined, high-fat diet (CDAHFD) feeding. The mRNA expression of *Nfkbiz* in the liver significantly augmented in wild-type C57BL/6 mice after CDAHFD (Fig. 1A). We further assessed the expression of *NFKBIZ* in the human liver and found a significant increase in *NFKBIZ* in moderate steatosis and a decrease in bridging fibrosis (Fig. 1B). To examine the role of IκBζ on the progression of NAFLD, we generated hepatocyte-specific IκBζ-deficient mice. *Nfkbiz*^*fl*/*fl*^ mice were crossed with *Alb-Cre* transgenic mice (*Alb-Cre;Nfkbiz*^*fl/fl*^). Male *Alb-Cre;Nfkbiz*^*fl/fl*^ mice and littermate male control *Nfkbiz*^*fl*/*fl*^ mice were fed with CDAHFD for 1–8 weeks to induce NAFLD. Robust mRNA expression of *Nfkbiz* in the liver was observed in *Nfkbiz*^*fl*/*fl*^ (Fig. 1C). An increase of *Nfkbiz* mRNA in the liver was not observed in *Alb-Cre;Nfkbiz*^*fl/fl*^ after CDAHFD challenge (Fig. 1C), which suggests that the induction of *Nfkbiz* in the liver during NAFLD development was mainly derived from albumin-producing hepatocytes; thus, other cells, including Kupffer cells, endothelial cells, and hepatic stellate cells, were not associated with the increase in IκBζ in this challenge.

### *Alb-Cre; Nfkbiz*^*fl/fl*^ mice accelerate the progression of NAFLD

We next examined liver morphological and histological analyses after CDAHFD feeding. An obvious white color change in the liver surface was observed in *Nfkbiz*^*fl*/*fl*^ mice at 8 weeks after CDAHFD (Fig. 1D). On the other hand, the progression of NAFLD demonstrated by the irregular changes on the liver surface was faster in *Alb-Cre;Nfkbiz*^*fl/fl*^ mice fed with CDAHFD (Fig. 1D). Approximately half of *Alb-Cre;Nfkbiz*^*fl/fl*^ mice developed an irregular surface at 8 weeks after CDAHFD feeding (data not shown). After the CDAHFD challenge, *Alb-Cre*;*Nfkbiz*^*fl/fl*^ mice had lighter livers than *Nfkbizfl/fl* mice (Supplemental Fig. 1). Furthermore, a significant increase in the plasma aspartate aminotransferase (AST), AST/alanine aminotransferase ratio, total bilirubin, and total bile acids were observed in *Alb-Cre;Nfkbiz*^*fl/fl*^ mice (Supplemental Fig. 1). In addition, the total protein was significantly decreased in *Alb-Cre; Nfkbiz*^*fl/fl*^ mice (Supplemental Fig. 1). We further compared histological changes in the liver after CDAHFD feeding by the nonalcoholic steatohepatitis score (NAS). *Alb-Cre; Nfkbiz*^*fl/fl*^ mice tended to have accelerated steatosis, inflammation, and ballooning after the CDAHFD diet (Fig. 1E,F, Supplemental Fig. 2). The mere expression of Cre in hepatocytes did not seem to accelerate the progression of NAFLD, because early lipid deposition was similar in the liver of *Alb-Cre* mice and C57BL/6 mice (Supplemental Fig. 3).

### Role of hepatocyte IκBζ for apoptosis, inflammation, and fibrosis during NAFLD development

**Figure 2:**
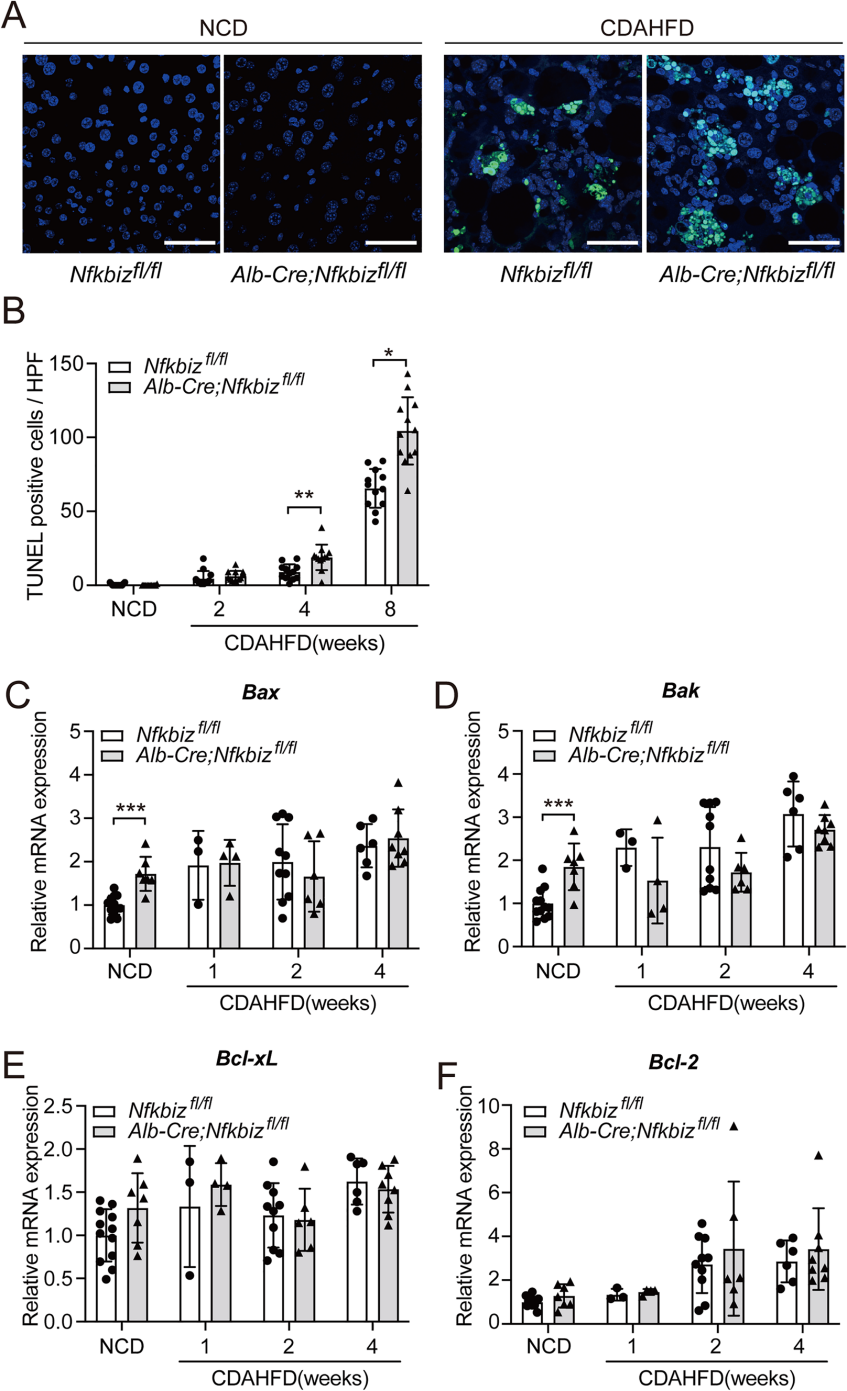
Deficiency of IκBζ in hepatocytes increases liver apoptotic cells in mouse model of NAFLD. *Nfkbiz*^*fl/fl*^ and *Alb-Cre; Nfkbiz*^*fl/fl*^ mice were fed an NCD or CDAHFD. (**A**) TUNEL staining of the liver section was observed using a confocal microscope (Leica TCS SP8; Leica Microsystems). Representative image of TUNEL staining of liver sections from mice fed an NCD or CDAHFD for 8 weeks. Green, apoptotic cells; Blue, DAPI. Bar = 50 µm. (**B**) The mean number of apoptotic cells in one section at high magnification from mice fed CDAHFD for 2, 4, or 8 weeks or NCD (white bar, *Nfkbiz*^*fl/fl*^; black bar, *Alb-Cre; Nfkbiz*^*fl/fl*^). Values are expressed as the mean ± SD (four independent fields per mouse, three mice per experiment). (**C, D, E, F**) mRNA expression of *Bax* (**C**), *Bak* (**D**), *Bcl-xl* (**E**), and *Bcl-2* (**F**) in the liver was assessed using real-time RT-PCR and was expressed as the fold increase in the *Nfkbiz*/*Hprt1* ratio (white bar, *Nfkbiz*^*fl/fl*^; gray bar, *Alb-Cre; Nfkbiz*^*fl/fl*^). Values are expressed as the mean ± SD (n = 3–12). CDAHFD, choline-deficient, L-amino acid-defined, high-fat diet; NCD, normal chow diet.*P < 0.05, **P < 0.01, ***P < 0.001.

Since the loss of IκBζ increased cell apoptosis in epithelial cells, which led to lymphocyte accumulation^26^, we next examined the signaling related to apoptosis. TUNEL staining at 4 and 8 weeks after CDAHFD feeding indicated an increase in apoptotic cells in the liver of *Alb-Cre;Nfkbiz*^*fl/fl*^ mice (Fig. 2A,B, Supplemental Fig. 4). Statistical significance between *Alb-Cre;Nfkbiz*^*fl/fl*^ mice and *Nfkbiz*^*fl/fl*^ mice was not observed at 2 weeks (Fig. 2B). We next examined the mRNA expression of the representative molecules to assess the state of apoptosis in the liver. The mRNA expressions of *Bax* and *Bak*, which are two nuclear-encoded mediators for apoptosis, were elevated in liver obtained from *Alb-Cre;Nfkbiz*^*fl/fl*^ mice fed with a commercial normal chow diet (NCD) (Fig. 2C,D). However, we could not detect significant changes in the *Bax* and *Bak* expressions between *Alb-Cre;Nfkbiz*^*fl/fl*^ mice and *Nfkbiz*^*fl/fl*^ mice fed with CDAHFD (Fig. 2C,D). There was no difference in the expressions of *Bcl-xL* and *Bcl-2*, which are negative regulators of *Bax* and *Bak* (Fig. 2E,F). We further investigated the expressions of apoptosis-related proteins by protein array analysis. We did not observe significant differences in the protein expressions of the liver at 4 weeks after the CDAHFD diet between *Alb-Cre;Nfkbiz*^*fl/fl*^ mice and *Nfkbiz*^*fl/fl*^ mice (Supplemental Fig. 5). We next examined ductular reaction after CDAHFD challenge because of the potential role of IκBζ in epithelial cell apoptosis. We assessed ductular reaction by Cytokeratin 19 (CK19) staining in liver specimens obtained from *Nfkbiz*^*fl/fl*^ and *Alb-Cre; Nfkbiz*^*fl/fl*^ mice and found no significant difference between groups (Supplemental Fig. 6).

**Figure 3:**
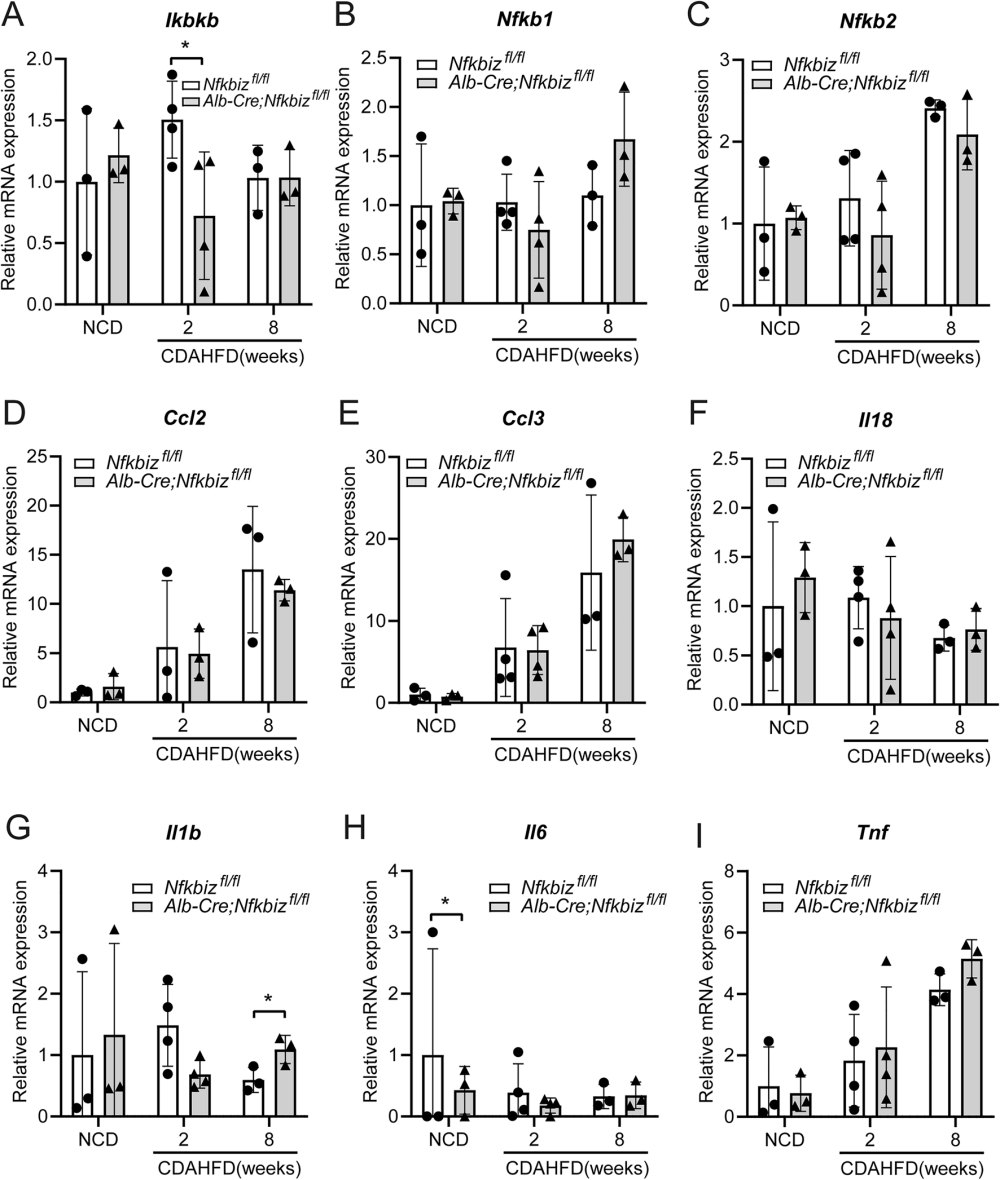
The deficiency of IκBζ in hepatocytes fails to modify mRNA expression of the proteins related to immune responses in mice model of NAFLD. *Nfkbiz*^*fl/fl*^ and *Alb-Cre; Nfkbiz*^*fl/fl*^ mice were fed an NCD or CDAHFD for 8 weeks. Liver RNA was isolated from mice at 2 and 8 weeks after the CDAHFD challenge or at 8 weeks after NCD. mRNA expressions of *Ikbkb* (**A**), *Nfkb1* (**B**), *Nfkb2* (**C**), *Ccl2* (**D**), *Ccl3* (**E**), *Il18* (**F**), *Il1b* (**G**), *Il6* (**H**), and *Tnf* (**I**) were analyzed by TaqMan Array Mouse Immune Panel, according to the manufacturer’s instructions (white bar, *Nfkbiz*^*fl/fl*^; gray bar, *Alb-Cre; Nfkbiz*^*fl/fl*^). Values are expressed as the mean ± SD (n = 3–4). *P < 0.05. CDAHFD, choline-deficient, L-amino acid-defined, high-fat diet; NCD, normal chow diet.

The development of NAFLD is characterized by hepatocyte apoptosis and the resultant inflammation and fibrosis. Since IκBζ is essential for the induction of secondary response genes via various IL and TLR responses, we analyzed mRNA expressions related to immune responses during NAFLD development with a TaqMan™ Array Mouse Immune Panel. Increased mRNA expressions of *Ccl2*, *Tnf*, and *Nfkb2* in the liver were observed with the progression of NAFLD (Fig. 3A–I) (*Ccl2, P* = 0.028 in *Nfkbiz*^*fl/fl*^; *Tnf*, *P* = 0.017 in *Nfkbiz*^*fl/fl*^; *Nfkb2*, *P* = 0.025 in *Nfkbiz*^*fl/fl*^). However, we did not detect significant differences in these mRNA expressions of the liver between *Alb-Cre;Nfkbiz*^*fl/fl*^ mice and *Nfkbiz*^*fl/fl*^ mice after being fed the CDAHFD diet (Fig. 3A–I). These data indicate that the deterioration of NAFLD in *Alb-Cre;Nfkbiz*^*fl/fl*^ mice is not attributable to transcriptional modification in apoptosis and the immune response.

**Figure 4:**
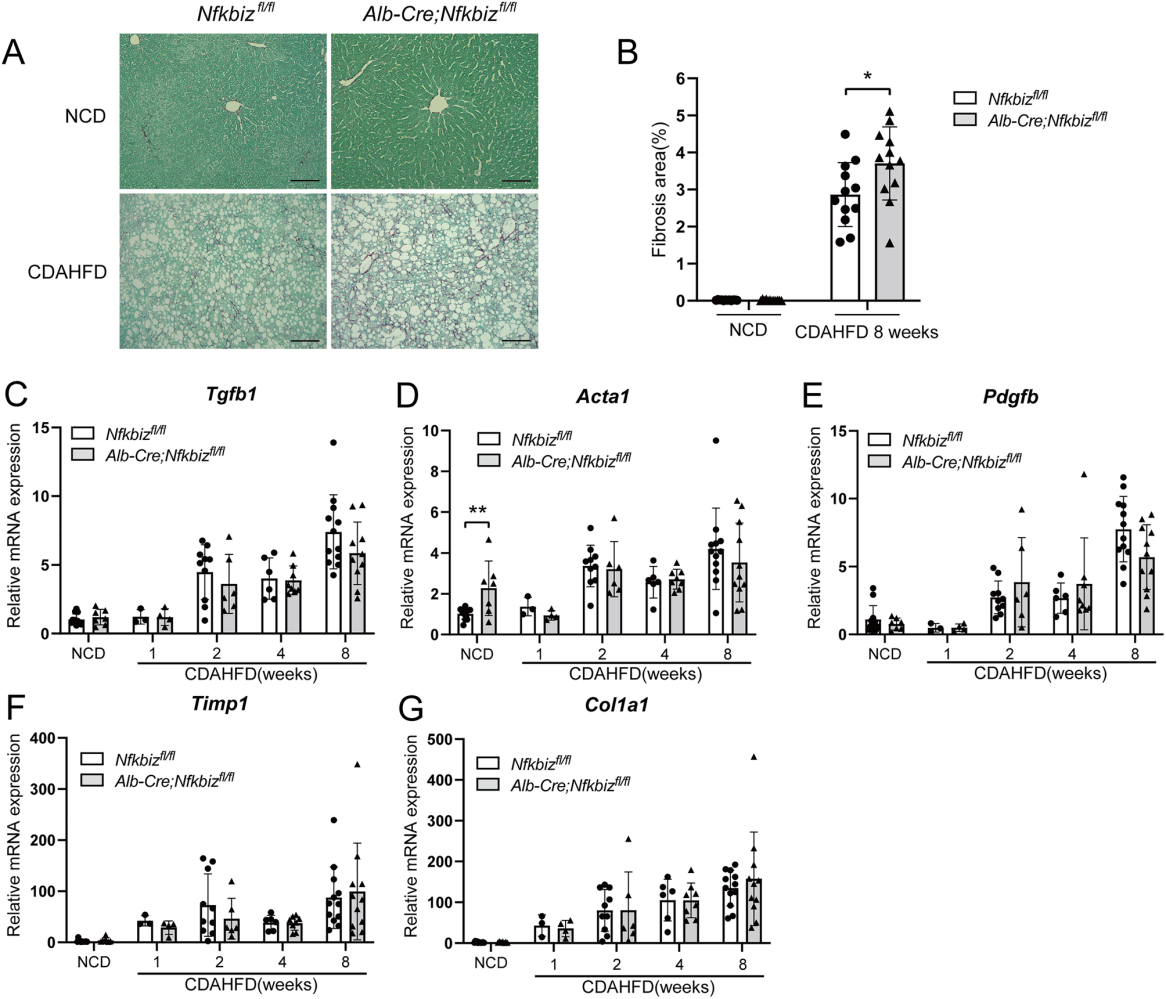
Fibrotic changes in the liver in the mice model of NAFLD. *Nfkbiz*^*fl/fl*^ and *Alb-Cre; Nfkbiz*^*fl/fl*^ mice were fed an NCD or CDAHFD for 8 weeks. (**A**) Representative image of Sirius Red staining of the liver obtained from *Nfkbiz*^*fl/fl*^ and *Alb-Cre; Nfkbiz*^*fl/fl*^ mice fed an NCD or CDAHFD for 8 weeks. Bar = 100 µm.** (B)** Sirius Red-positive area quantified using BZ-X 700 imaging software (Keyence). The fibrosis area was expressed as the percentage of the Sirius Red-positive area. Values are expressed as the mean ± SD (four independent fields per mouse, three mice per experiment). (**C–G**) mRNA expressions of *Tgfb1* (**C**), *Acta1* (**D**), *Pdgfb* (**E**), *Timp1* (**F**), and *Col1a1* (**G**) were analyzed by real-time RT-PCR and expressed as the fold increase in the *Nfkbiz*/*Hprt1* ratio (white bar, *Nfkbiz*^*fl/fl*^; gray bar, *Alb-Cre; Nfkbiz*^*fl/fl*^). Values are expressed as the mean ± SD (n = 3–12). *P < 0.05. CDAHFD, choline-deficient, L-amino acid-defined, high-fat diet; NCD, normal chow diet.

We next assessed fibrosis and mRNA expression of the related proteins after CDAHFD challenge. Histological analysis to observe fibrosis by Sirius Red staining revealed an increase in the fibrosis area after CDAHFD feeding in *Alb-Cre;Nfkbiz*^*fl/fl*^ mice (Fig. 4A,B). Increased mRNA expression of *Tgfb1*, *Acta1*, *Pdgfb*, *Timp1*, and *Col1a1* in the liver were observed with the progression of NAFLD (Fig. 4C–G) (*P* < 0.001 in *Nfkbiz*^*fl/fl*^). Although the expression of *Acta1* in *Alb-Cre; Nfkbiz*^*fl/fl*^ mice increased compared with *Nfkbiz*^*fl/fl*^ in the NCD-fed mice, we did not detect significant differences in the mRNA expression in the liver between the groups after being fed the CDAHFD diet (Fig. 4C–G).

### Role of hepatocyte IκBζ for hepatic steatosis in NAFLD

**Figure 5:**
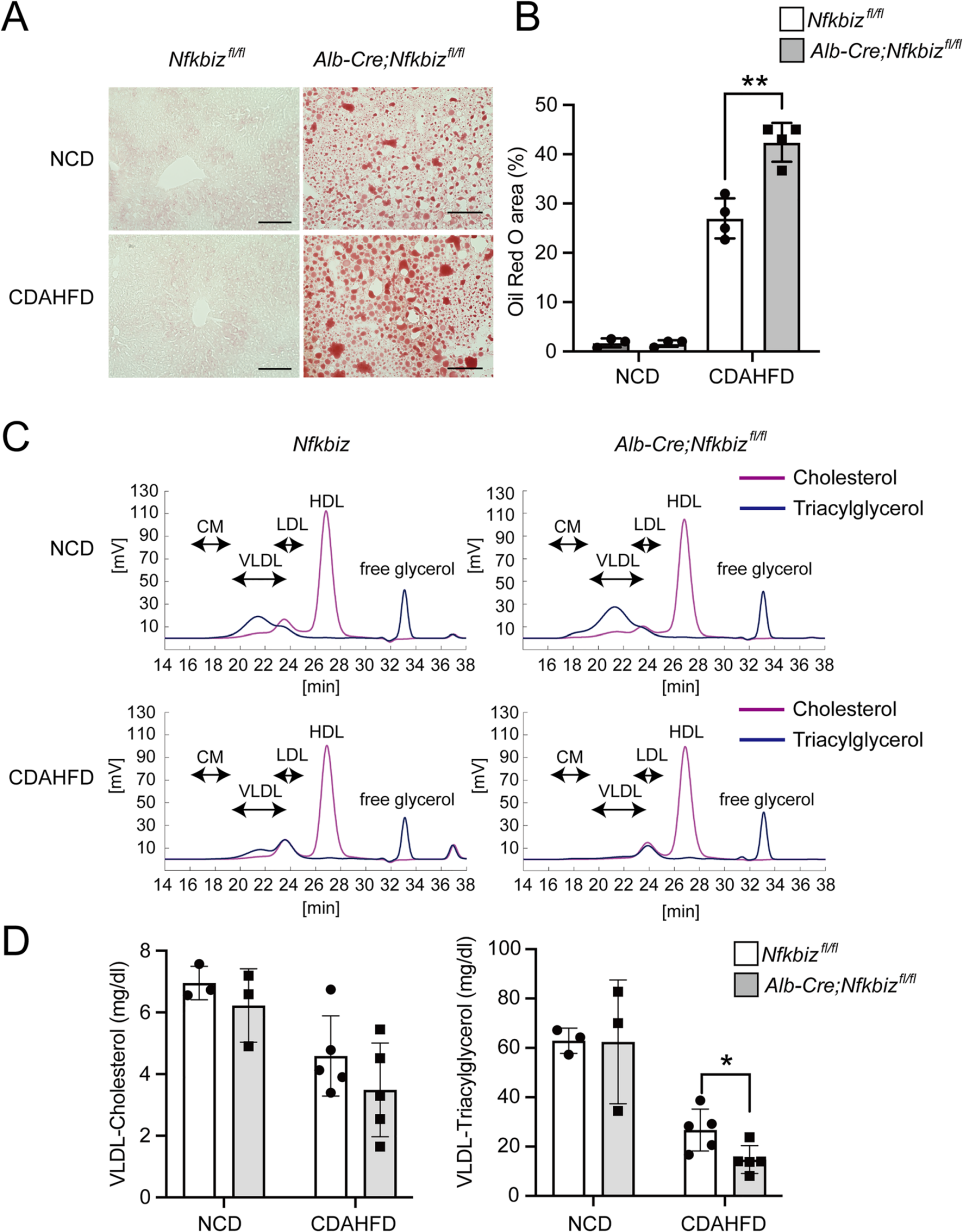
Deficiency of IκBζ in hepatocytes increases liver steatosis cells in the mouse model of NAFLD. *Nfkbiz*^*fl/fl*^ and *Alb-Cre; Nfkbiz*^*fl/fl*^ mice were fed an NCD or CDAHFD for 2 weeks. (**A**) Representative image of Oil Red O staining of liver sections from mice fed an NCD or CDAHFD for 2 weeks. Bar = 100 µm. (**B**) Oil Red O staining area of liver specimens quantified using BZ-X 700 imaging software (Keyence). The Oil red O staining area was expressed as the percentage of the Oil Red O area. Values are expressed as the mean ± SD (n = 4). (**C, D**) Plasma lipoprotein fractions were analyzed by high-performance liquid chromatography. (**C**) Representative image of the chromatogram (violet line, cholesterol; blue line, triacylglycerol). CM, chylomicron; VLDL, very low-density lipoprotein; LDL, low-density lipoprotein; HDL, high-density lipoprotein. (**D**) VLDL-cholesterol and VLDL-triacylglycerol in plasma were quantified (white bar, *Nfkbiz*^*fl/fl*^; gray bar, *Alb-Cre; Nfkbiz*^*fl/fl*^). Values are expressed as the mean ± SD (n = 3–5). *P < 0.05, **P < 0.01. CDAHFD, choline-deficient, L-amino acid-defined, high-fat diet; NCD, normal chow diet.

Since *Alb-Cre;Nfkbiz*^*fl/fl*^ accelerated the progression of NAFLD at an early stage, we hypothesized that lipid accumulation in the hepatocytes may have enhanced after CDAHFD challenge in *Alb-Cre;Nfkbiz*^*fl/fl*^ mice. We used histological analysis to evaluate lipid accumulation in the liver of *Alb-Cre;Nfkbiz*^*fl/fl*^ mice and *Nfkbiz*^*fl/fl*^ mice fed with NCD or CDAHFD for 2 weeks. The steatosis in the liver assessed by Oil Red O staining significantly increased in *Alb-Cre;Nfkbiz*^*fl/fl*^ mice (Fig. 5A,B), which suggests that hepatocyte IκBζ may contribute to NAFLD progression via aberrant lipid metabolism. Choline-deficient diets exacerbated steatosis by inhibiting very low-density lipoprotein (VLDL) release from the liver^28^. Indeed, the plasma VLDL concentration was significantly decreased by the CDAHFD diet (Fig. 5C,D). Deletion of IκBζ in hepatocytes further reduced plasma VLDL-derived triacylglycerol after the CDAHFD challenge (Fig. 5C,D). Although the levels of high-density lipoprotein (HDL)-derived cholesterol tend to be reduced by deletion of IκBζ in hepatocytes, it was not significant after the CDAHFD challenge (Supplemental Fig. 7). Changes in LDL cholesterol were not observed (Supplemental Fig. 7).

### Overexpression of IκBζ in hepatocytes by the AAV8 vector attenuates liver steatosis

**Figure 6:**
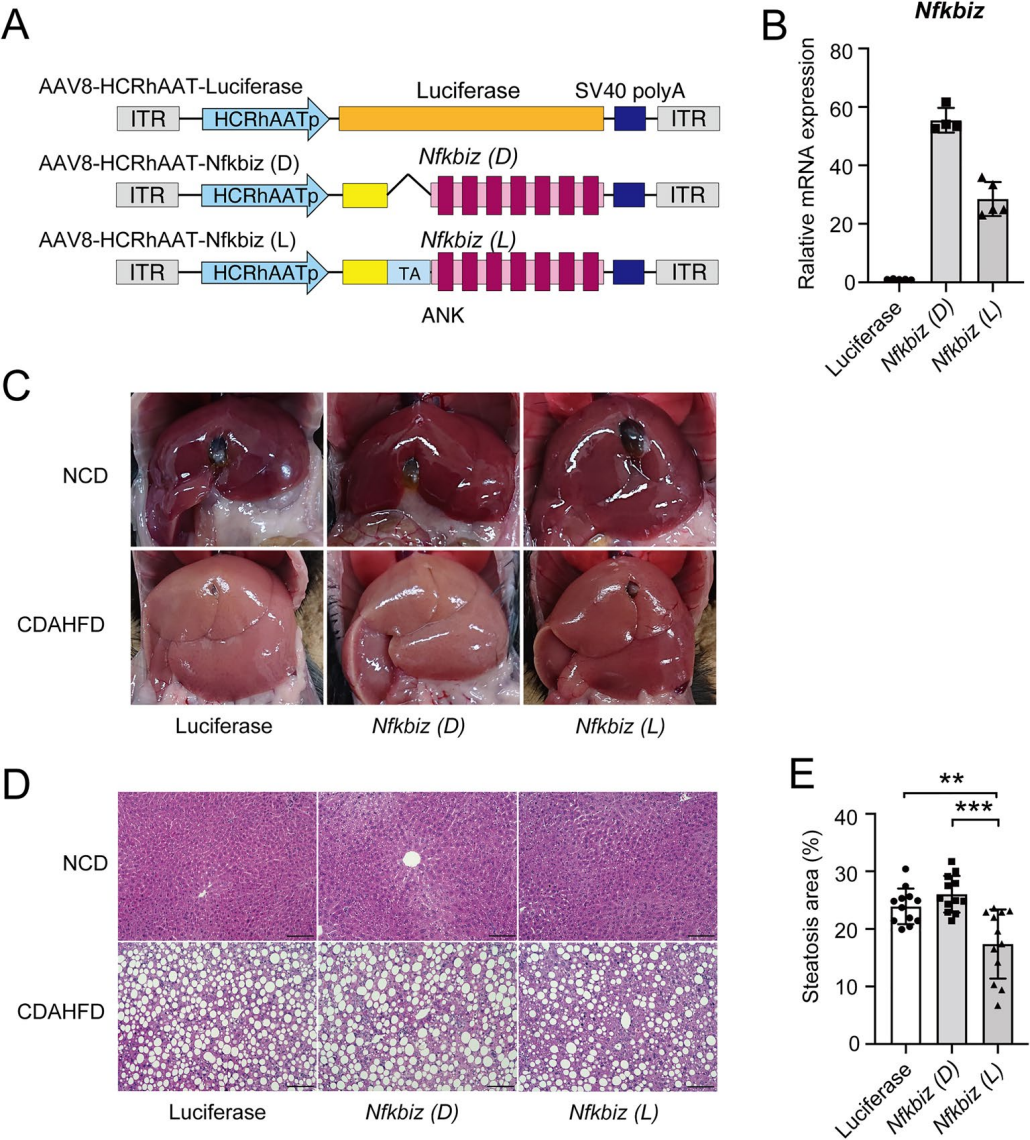
Overexpression of *Nfkbiz* in liver hepatocytes by AAV8 vector ameliorates hepatic steatosis in vivo. (**A**) Schematic representation of the AAV8 vector used in this study. *Nfkbiz (D)*, a deletion mutant of IκBζ lacking trans-activating domain; *Nfkbiz (L)*, full-length IκBζ; ITR, internal terminal repeat; ANK, ankyrin repeat. (**B**) C57BL/6 mice (7 weeks old) were intravenously treated with 3 × 10^11^ vg of AAV8 vector harboring *Luciferase*, *Nfkbiz* (D), or *Nfkbiz* (L). The expression of *Nfkbiz* in the liver obtained at 4 weeks after vector injection was assessed to measure the mRNA of *Nfkbiz* using real-time PCR and was expressed as the fold increase in the *Nfkbiz*/*Hprt1* ratio. Values are expressed as the mean ± SD (n = 4–5). (**C–E**) At 4 weeks after AAV injection, the mice were then fed an NCD or CDAHFD for 4 weeks. (**C**) Representative morphology of the liver. (**D)** Representative image of hematoxylin–eosin staining of the liver section. Bar = 100 µm. (**E**) Steatosis area in the section quantified with BZ-X 700 imaging software (Keyence). The steatosis area was expressed as a percentage of the fat droplet area. Values are expressed as the mean ± SD (four independent fields per mouse, three mice per experiment). **P < 0.01, ***P < 0.001. AAV, adeno-associated virus; CDAHFD, choline-deficient, L-amino acid-defined, high-fat diet; NCD, normal chow diet.

We next examined whether overexpression of IκBζ attenuates hepatic steatosis in vivo. We constructed three types of AAV8 vectors to express target proteins to liver hepatocytes; (1) firefly *Luciferase* (control); (2) *Nfkbiz* (L), a full-length mouse IκBζ; (3) *Nfkbiz* (D), truncated form of IκBζ without transcriptional activity^26^ (Fig. 6A). AAV8 vectors harboring *Luciferase*, *Nfkbiz* (L), and *Nfkbiz* (D) were intravenously injected into C57BL/6 wild-type mice. We confirmed overexpression of *Nfkbiz* in the liver at 4 weeks after the vector injection (Fig. 6B). The treated mice were further fed with CDAHFD for 4 weeks. There was no significant difference in the liver weight (data not shown) among the three groups, but the white color change of the liver due to the CDAHFD diet was significantly abolished by the hepatocyte expression of *Nfkbiz* (L), but not *Nfkbiz* (D) (Fig. 6C). Histological examination confirmed the inhibition of liver steatosis by the expression of *Nfkbiz* (L) (Fig. 6D,E). In contrast, hepatic apoptosis and fibrosis were not ameliorated by the expression of *Nfkbiz* (L) (Supplemental Fig. 8).

### Changes in the transcript expressions in hepatocytes due to overexpression of IκBζ

**Figure 7:**
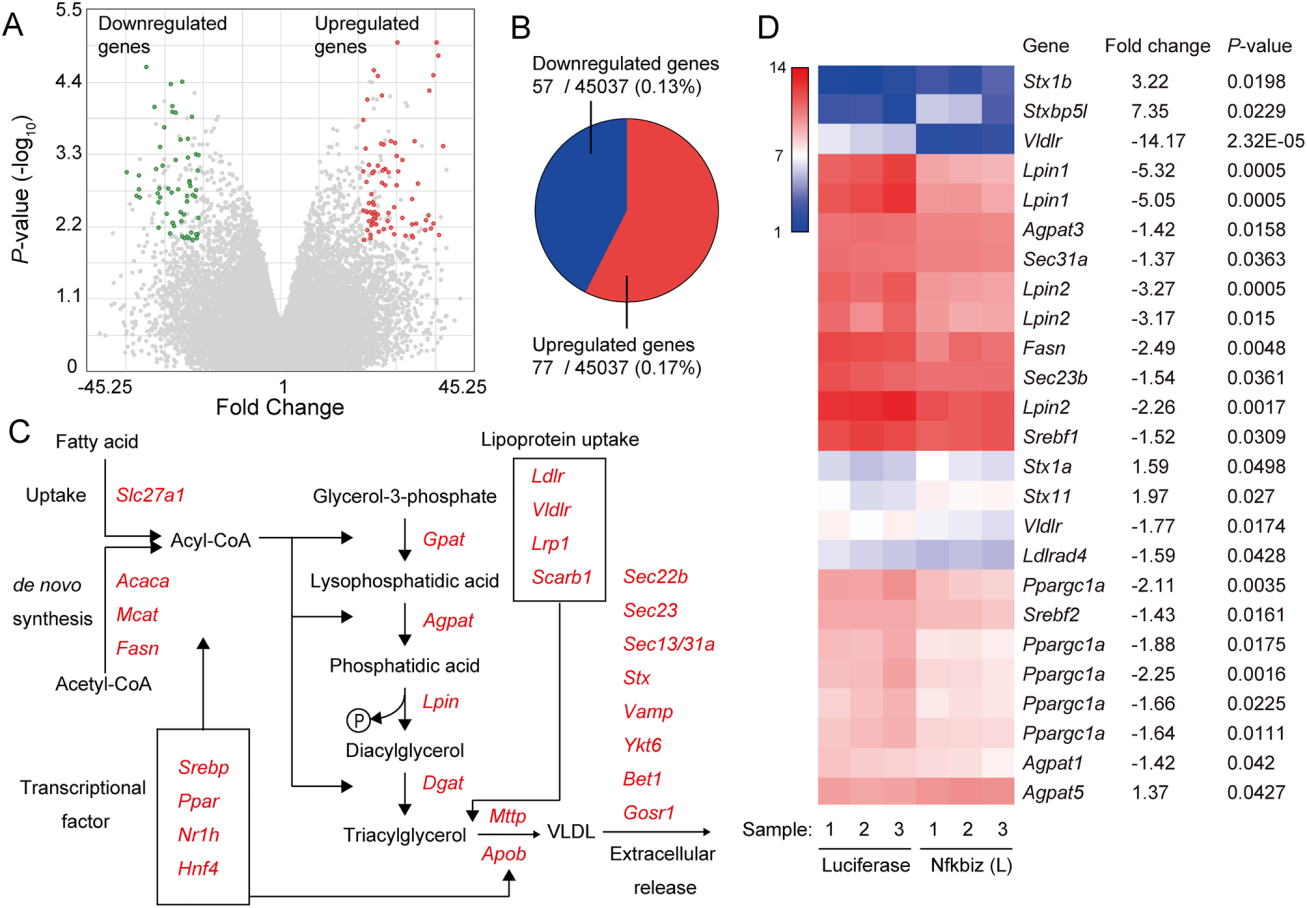
Microarray analysis in the liver of mice treated with the AAV8 vector harboring *Nfkbiz*. C57BL/6 mice (7 weeks old) were intravenously treated with 3 × 10^11^ vg of the AAV8 vector harboring *Luciferase* or *Nfkbiz* (L). The CDAHFD challenge started at 4 weeks after vector injection. Liver RNA was isolated from mice at 4 weeks after the challenge. The expressions of transcripts in the liver were analyzed by Mouse Genome 430 2.0 Array (n = 3 in each group). The gene expressions in the liver of mice treated with the AAV vector expressing *Nfkbiz* (L) were compared with those treated with the AAV vector expressing *Luciferase*. (**A**) Volcano plots showing the global transcriptional changes among the groups. All genes on the Mouse Genome 430 2.0 Array were plotted. The log-fold changes in *Nfkbiz* (L) versus *Luciferase* group are represented on the X-axis. Statistically upregulated and downregulated genes are expressed in red and green, respectively (> fivefold, *P* < 0.01). (**B**) The proportion of upregulated and downregulated genes. (**C**) Overview of triacylglycerol metabolism in the liver. Transcripts selected for the analysis are shown in red italics. (**D**) Transcripts related to triacylglycerol metabolism exhibit statistically significant changes (*P* < 0.05). The fold changes and *P* values are shown on the right.

To identify the mechanisms by which the expression of IκBζ attenuates liver steatosis, we compared mRNA expressions in liver obtained from mice treated with the AAV vector harboring *Nfkbiz* with those treated with the AAV vector expressing *Luciferase* by microarray analysis. We analyzed a set of 45,037 probes to detect the mRNA expression and selected genes whose expression changed more than fivefold (*P* < 0.01) in liver obtained from mice treated with AAV8 vector harboring *Nfkbiz* (Fig. 7A,B). We identified 77 upregulated genes and 57 downregulated genes (Fig. 7B, Supplemental Fig. 9, Supplemental Tables 2 and 3). Since we noticed the downregulation of Lipin (*Lpin*), which is a catalyzing enzyme for the synthesis of glycolipids, we extracted genes related to triacylglycerol metabolism to synthesis VLDL in the liver (Fig. 7C, Supplemental Fig. 9).

Of mRNAs related to fatty acid uptake and de novo synthesis (Fig. 7C), the expressions of fatty acid synthetase (*Fasn*) and transcriptional factors that regulate fatty acid synthesis including sterol regulatory element-binding protein-1 (SREBP-1, *Srebf 1*), SREBP-2 (*Srebf 2*), peroxisome proliferator-activated receptor (PPARγ) coactivator 1α (*Prargc1a*) were attenuated by the expression of IκBζ (Fig. 7D). The glycerol phosphate pathway is the major pathway by which triacylglycerol is generated in the liver (Fig. 7C). The expressions of some acyltransferases (*Agpat*1 and *Agpat3*), as well as the phosphatases Lipin1 (*Lpin1*) and Lipin2 (*Lpin2*), were significantly downregulated (Fig. 7D). PPARγ coactivator 1α (*Ppargc1a*), which is a transcriptional coactivator controlling several key hepatic metabolic pathways, was also downregulated. Triacylglycerol was further assembled with Apoprotein B (*ApoB*) by the microsomal triglyceride transfer protein (*Mttp*) and secreted into circulation as a VLDL by the vesicular transport (Fig. 7C). We also observed increases in the syntaxin family members involved in vesicular transport, including *Stx1b*, *Stx1a,* and *Stx11,* and the downregulation of *Sec23b* and *Sec 31a* (Fig. 7D). Furthermore, the expression of the VLDL receptor (*Vldlr*) was downregulated by the expression of IκBζ. Among the changes observed in the microarray analysis, we confirmed the significant decrease of *Lipin1* mRNA expression by performing RT-PCR in mice treated with AAV vector harboring *Nfkbiz*. (Supplemental Fig. 10). Next, we assessed the changes in the transcripts related to β-oxidation by overexpression of IκBζ. As shown in Supplemental Table 4, no significant change was observed in the transcripts.

## Discussion

Inflammation is important for the process of NAFLD, from hepatic lipid accumulation to liver fibrosis^28,29^. TLRs and ILs, which are involved in innate immunity, have been reported to promote the progression of NAFLD^30^. TLRs and ILs induce inflammation through transcriptional regulation via NF-κB^17^. IκBζ is a transcription factor that amplifies signals downstream of NF-κB, thus leading to a secondary inflammatory response^31–33^. In this study, we generated hepatocyte-specific IκBζ-deficient mice to clarify the role of IκBζ in NAFLD progression. Contrary to our initial expectation, the deletion of IκBζ in hepatocytes accelerated the progression of NAFLD. These results suggest the existence of a novel function of IκBζ in regulating the progression of NAFLD in hepatocytes.

Our data suggest that IκBζ regulates the progression of NAFLD by controlling intracellular triglyceride accumulation in hepatocytes. IκBζ-deficient mice showed enhanced hepatic fibrosis in CDAHFD-induced NAFLD. We analyzed the processes leading to fibrosis, including inflammation, apoptosis, and fat accumulation, to evaluate the influence of IκBζ deficiency. Since the expression of inflammatory cytokines and chemokines were not different in IκBζ-deficient mice after the CDAHFD challenge, it is unlikely that IκBζ directly modulates the inflammatory processes in hepatocytes. Our experiments employed the hepatocyte-specific deletion of IκBζ; however, the role of IκBζ in blood cells for inflammation may be negligible. The deletion of IκBζ was previously reported to be associated with the induction of apoptosis in epithelial cells^26^. Although apoptosis-inducing factors, such as *Bax* and *Bak*, were predominantly expressed in the liver in IκBζ-deficient mice fed with a normal diet, the enhancement of apoptosis induction is unlikely, because there was no significant difference in apoptosis-related protein or gene expressions during the CDAHFD challenge. On the other hand, lipid accumulation in hepatocytes, with the earliest change appearing in NAFLD, was significantly enhanced in IκBζ-deficient mice, and overexpression of IκBζ suppressed fat accumulation in NAFLD in wild-type mice. Furthermore, moderate, but not bridging fibrosis enhanced the expression of *NFKBIZ*, suggesting that *NFKBIZ* plays a role in the early stage of NAFLD. This data supports our hypothesis that IκBζ regulates lipid metabolism in hepatocytes.

IκBζ regulation of fat accumulation in hepatocytes may be partly explained by the involvement in VLDL release from the liver. CDAHFD promotes lipid accumulation by inhibiting the release of VLDL from the liver due to choline deficiency. In fact, CDAHFD reduced the concentration of VLDL-triacylglycerols in the blood. The concentration of plasma VLDL-triacylglycerol was more suppressed in IκBζ-deficient mice, and the expressions of some syntaxin members related to vesicular transport were upregulated by the expression of IκBζ. However, this activity alone does not clearly explain the apparent fat accumulation in the liver, because the reduction of plasma VLDL-triacylglycerol was only 18.8% (*Nfkbiz*^*fl/fl*^ 57.6% *v.s. Alb-Cre;Nfkbiz*^*fl/fl*^ 76.4%). The expression of IκBζ lacking a transcription factor from the AAV vector showed no inhibitory effect on lipid accumulation, which indicates that IκBζ plays a role in the regulation of lipid metabolism through its transcriptional activity.

We found that IκBζ may affect mRNA expression of genes associated with the triglyceride synthesis pathway. The synthesis of triglycerides involves three fatty acid-derived acyl-CoA esterified to a glycerol molecule^34^. Fatty acids are synthesized de novo from acetyl CoA and are taken up from extracellular sources^35^. Microarray analysis showed the downregulation of the molecules related to this process. Notably, we confirmed the downregulation of the *Lipin1* by IκBζ expression. Lipin1 plays a key role in orchestrating liver metabolism, for example by generating diacylglycerol through its phosphatidate phosphatase activity^36^. Lipin1 also acts as a transcriptional coactivator for PPARα and PGC1α, inducing the expression of genes related to fatty acid oxidation^36^. Besides, liver-specific deletion in mice aggravates lipid accumulation in the liver by inhibiting of VLDL excretion and fatty acid oxidation in alcoholic liver injury^37^. In addition, hypoxia-inducible factor-1 prevents hepatic lipid accumulation, probably by regulating Lipin1 and PGC1α ^38^. In this study, the overexpression of IκBζ ameliorated fat accumulation in the liver despite the decrease in *Lipin1*. This discrepancy may involve the induction of a negative regulator or a compensatory mechanism for the expression of some factors to ameliorate steatosis through the expression of IκBζ. In the future, it will be necessary to study in more detail to identify a direct target that improves fat accumulation in the liver through the transcriptional activation of IκBζ.

It would be of clinical importance to understand the relationship between IκBζ regulation of lipid metabolism in the liver and the function of other organs, as well as the role of IκBζ in the metabolism of other tissues. NAFLD is closely related to the development of type 2 diabetes mellitus^39,40^. Insulin resistance observed in type 2 diabetes exacerbates NAFLD by increasing fatty acid influx and fatty acid synthesis in the liver^41^. Conversely, NAFLD promotes insulin resistance, which leads to exhaustion of pancreatic β cells and exacerbates diabetes^42^. Interestingly, overexpression of IκBζ in wild-type mouse hepatocytes with the AAV vector not only suppressed the development of NAFLD, but also caused weight gain (data not shown). Although the mechanism for this is not clear at present, it is possible that the regulation of lipid metabolism by IκBζ in hepatocytes is linked to a systemic metabolic process to regulate body weight. Furthermore, it would be interesting to investigate the function of IκBζ in other organs involved in lipid metabolisms, such as adipose tissue and muscle. Detailed analyses about the role of IκBζ in systemic lipid metabolism may provide insights into whether IκBζ is a new target for hyperlipidemia drugs.

This study has several limitations that should be addressed. First, the precise mechanism of hepatic lipid metabolism regulated by IκBζ has not been fully elucidated. IκBζ reportedly facilitated the transcription of a set of inflammatory genes, but IκBζ overexpression without transcriptional activity failed to ameliorate steatosis. Although it is certain that the transcriptional regulation controls fat accumulation in the liver, we could not identify key molecules regulated by IκBζ. Second, we observed tiny but significant differences between NCD-fed *Alb-Cre; Nfkbiz*^*fl/fl*^ and *Nfkbiz*^*fl/fl*^ mice. For example, NCD-fed *Alb-Cre; Nfkbiz*^*fl/fl*^ mice had higher body and liver weight (Supplemental Fig. 1). We noted similar differences in serum AST, total bilirubin, and mRNA expressions of *Bax* and *Bak* (Fig. 2). A deficiency of *Nfkbiz* in liver hepatocytes may harm liver function and the metabolic system, even in mice on a long-term NCD. Furthermore, the present study was mainly conducted in a mouse NAFLD model using CDAHFD. Although it is histologically similar to human steatohepatitis, it does not cause weight gain or insulin resistance observed in human^43^. We also used only male mice in this study, and thus sex difference in the role of IκBζ in the development of NAFLD are unknown. Finally, our results should be interpreted with caution because the study was conducted only on mice. Therefore, it is unknown whether IκBζ has a similar function in the development and progression of human NAFLD, albeit we observed the increase in *NFKBIZ* in moderate steatosis. Further analysis is required to determine whether the induction of IκBζ in the liver of human NAFLD patients regulates the progression of NAFLD.

In conclusion, we demonstrated that IκBζ contributes to the development of NAFLD through alterations in lipid metabolism in the liver. With the resolution of viral hepatitis by effective antihepatitis virus drugs and increases in obesity worldwide, the importance and prevalence of NAFLD is expected to further increase in the future. Further elucidation of the regulatory mechanism of steatosis by IκBζ may represent a novel and attractive therapeutic target for NAFLD, diabetes, and obesity. Further studies are required to elucidate the precise mechanisms by which IκBζ controls lipid metabolism so that an effective treatment that targets IκBζ can be developed for various diseases.

## Methods

### Animal experimentation

All animal experimental procedures were approved by The Institutional Animal Care and Concern Committee of Jichi Medical University (permission number: 17118-04), and animal care was conducted in accordance with the committee’s guidelines and ARRIVE guidelines. C57BL/6J mice were purchased from Japan SLC (Shizuoka, Japan). Mice containing *loxP-*flanked *Nfkbiz*^*fl/fl*^ were obtained from RIKEN BRC (#RBRC06410, Ibaraki, Japan)^26^. The deletion of both *Nfkbiz* alleles in hepatocytes was achieved by breeding *Nfkbiz*^*fl*/*fl*^ animals with *Alb-Cre* transgenic mice (B6.Cg-Tg(Alb-Cre)21Mgn/J)^44^. The PCR primer used for genotyping is shown in Supplemental Table 1. Hepatocyte-specific *Nfkbiz*-deleted male mice (*Alb-Cre:Nfkbiz*^*fl*/*fl*^) and a littermate control male mice (*Nfkbiz*^*fl*/*fl*^) were used for the experiments. Animals were maintained in isolators in the specific pathogen‐free facility of Jichi Medical University at 23 °C ± 3 °C with 12:12 h light/dark cycle and fed with a commercial normal chow diet (NCD) (CLEA Japan, Inc., Shizuoka, Japan) after weaning. We genotyped mice at 4 weeks of age, and littermates with the same genotype were maintained at a maximum of five mice per cage. To induce steatohepatitis, 7–8-week-old male mice were fed a choline-deficient, L-amino acid-defined, high-fat diet (CDAHFD) (60 kcal% lard-based fat, choline deficient, and 0.1% methionine) (A06071302, Research Diets Inc., New Brunswick, NJ)^45^. When indicated, whole blood was obtained from anesthetized mice through the jugular vein using a 30 G syringe containing 1/10 sodium citrate, which was then centrifuged to obtain plasma samples. Clinical chemistry parameters were measured at Oriental Yeast Co. (Tokyo, Japan). The lipoprotein fraction was analyzed using high-performance liquid chromatography at Skylight Biotech Inc. (Akita, Japan).

### Quantitative reverse transcription polymerase chain reaction

Mice anesthetized with isoflurane were intracardially perfused with PBS to remove blood, and then liver tissues were sterilely isolated. The tissues were incubated with RNAlater®Stabilizaion Reagent (QIAGEN, Venlo, Netherlands) at 4 °C overnight, and then stored at − 80 °C in a deep freezer until extraction. Total RNA was isolated from cells using an RNeasy Plus Mini Kit (Qiagen). We purchased RNAs of human liver from healthy, moderate steatosis, and steatohepatitis with bridging fibrosis (SEKISUI Medical Co., Tokyo, Japan; normal liver: #Lot. H1314, macro fat: 0%, 43 years old Caucasian male, body mass index (BMI): 30, alcohol use: none, diabetes: none; steatosis: moderate: #Lot. H0878, macro fat: 40%, 43 years old Caucasian male, BMI: 35.4, alcohol use: none, diabetes: none; steatohepatitis: bridging fibrosis: #Lot. H0613, macro fat: 50%, 65 years old Caucasian male, BMI: 32.9, alcohol use: none, diabetes: none). The RNA samples were reverse-transcribed using a PrimeScript RT Reagent kit (Takara Bio, Shiga, Japan) or SuperScript™ IV VILO™ Master Mix with ezDNase™ Enzyme (Thermo Fisher Scientific, Waltham, MA, USA). Quantitative real-time PCR was performed using THUNDERBIRD™ Probe qPCR Mix or THUNDERBIRD™ SYBR qPCR Mix (TOYOBO, Osaka, Japan) in QuantStudio™ 12 K Flex Real-Time PCR system (Thermo Fisher Scientific). Reactions were analyzed in duplicate, and expression levels were normalized to *Gapdh* or *Hprt1* mRNA levels (ΔCT). We then calculated ΔΔCT as the ratio of calibrator sample (an NCD-fed mouse). The calculation was automatically performed in QuantStudio™ 12 K Flex Real-Time PCR system (Thermo Fisher Scientific). The primers used in this study are shown in Supplemental Table 1. Quantitative gene expressions of targets associated with immune responses were analyzed by TaqMan™ Array Mouse Immune Panel (Thermo Fisher Scientific), according to the manufacturer’s instructions.

### Histological analysis for liver steatosis and fibrosis

Mice were anesthetized with isoflurane and then intracardially perfused with PBS to remove the blood. Then, the liver tissues were dissected and fixed in 10% formalin and embedded in paraffin or were fixed with 4% paraformaldehyde, incubated with PBS containing sucrose (10%–30%), and then frozen in the presence of optimal cutting temperature compound (Sakura Fintek Japan, Tokyo, Japan). The paraffin-embedded tissue sections were dewaxed in xylene, rehydrated with ethanol, and washed with water. The sections were processed for hematoxylin and eosin staining, Sirius Red staining (Sirius Red/Fast Green Collagen Staining Kit, Chondrex Inc., Woodinville, WA, USA), or immunostaining. The frozen sections were subjected to Oil Red O staining (FUJIFILM Wako Pure Chemical, Osaka, Japan). The sections were observed using an all-in-one microscope (BZ-X700, Keyence, Tokyo, Japan). Quantitative evaluation of the target area was measured with BZ-X 700 imaging software (Keyence). When indicated, the NAFLD activity score (NAS) in hematoxylin and eosin staining was assessed by a blinded researcher (Dr. Miura, Jichi Medical University), as previously described^4^. Hepatocellular steatosis was scored as 0 (< 5% of liver parenchyma), 1 (5%–33%), 2 (33%–66%), and 3 (> 66%). Lobular inflammation was also scored with hematoxylin and eosin staining as 0 (no inflammatory foci), 1 (< 2 foci per 20 × field), 2 (2–3 foci per 20 × field), and 3 (> 4 foci per 20 × field).

### Immunostaining

The sections were pretreated with 5% donkey serum and then treated with anti-Cytokeratin 19 (CK19) rabbit polyclonal antibody (GENETEX Inc., Irvine, CA). Immunoreactivity was detected with Simple Stain Mouse MAX-PO (Nichirei Bioscience, Tokyo, Japan), and DAB (Agilent Technologies, Santa Clara, CA, USA), followed by counterstaining with Mayer’s hematoxylin. Tissue sections were observed using an all-in-one microscope (BZ-X700, Keyence, Tokyo, Japan). Quantitative evaluation of the CK19-positive area was performed with BZ-X 700 imaging software (Keyence).

### TUNEL staining of apoptotic cells in the liver section

TUNEL staining of apoptotic cells in the liver sections was performed with Mebstain Apoptosis Tunel Kit III (Medical & Biological Laboratories Co, Aichi, Japan). Briefly, paraffin-embedded tissue sections were dewaxed in xylene and then rehydrated through immersion in ethanol followed by washing with water. After proteinase K treatment, the DNA nick ends were labeled with biotin-conjugated dUTP by terminal deoxynucleotidyl transferase and then stained with streptavidin conjugated with dichlorotriazinyl aminofluorescein. The sections were mounted in Vectashield Mounting Medium with DAPI (Vector Laboratories, Burlingame, CA, USA). Images were obtained using a fluorescence microscope (BZ-X700, Keyence) or a confocal microscope (Leica TCS SP8, Leica Microsystems, Wetzlar, Germany). Quantitative evaluation was determined as the apoptotic cell number of one section recorded at high magnification with BZ-X700 imaging software (Keyence).

### Expression of apoptosis-related proteins

Liver tissues were harvested in 500 µL PBS containing cOmplete® protease inhibitor cocktail (Sigma Aldrich, Saint Louis, MO, USA), sonicated, and then lysed with the addition of Triton X-100 (final concentration 1%). After the freeze–thaw cycle, samples were centrifuged at 10,000 × *g* for 5 min to remove cell debris. The relative levels of apoptosis-related protein in the supernatants (400 µg) were determined by a Proteome Profiler Mouse Apoptosis Array Kit (R&D Systems, Minneapolis, MN, USA), according to the manufacturer’s instructions. The image data were quantified using the ImageQuant LAS4000 system (GE Healthcare). Band intensities were normalized to that of the internal control.

### Construction of an adeno-associated virus vector

cDNAs of *Nfkbiz* [*Nfkbiz* (L) and (D)] were kindly provided by Dr. T. Maruyama (National Institutes of Health). A DNA fragment consisting of a chimeric promoter (HCRhAAT; an enhancer element of the hepatic control region of the Apo E/C1 gene and the human anti-trypsin promoter), full-length of *Nfkbiz*, truncate type of *Nfkbiz* without transcriptional activity, or *Luciferase* cDNA, and the SV40 polyadenylation signal were introduced between inverted terminal repeats into pAAV2. The AAV vectors were produced by triple plasmid transfection of human embryonic kidney 293 cells to generate the AAV8 vector (helper-free system), as described previously^46^. Titration of recombinant AAV vectors was carried out by quantitative PCR to measure the copy number of the SV40 polyadenylation signal.

### Microarray analysis

The total RNAs were isolated by RNeasy Mini Kit (QIAGEN). mRNA expressions were analyzed by GeneChip Mouse Genome 430 2.0 Array (Thermo Fisher Scientific) and detected by GeneChip Scanner 3000 7G (Thermo Fisher Scientific) at Takara Bio Co. The data were analyzed with Transcriptome Analysis Console software (Thermo Fisher Scientific).

### Statistical analysis

Data were analyzed using GraphPad Prism® version 8 (GraphPad Software, San Diego, CA, USA). All data are presented as the means ± standard deviation (SD). Statistical significance was determined using two-tailed Student’s *t*-tests. A value of *P* < 0.05 was considered to be statistically significant.

## Supplementary Information

Supplementary Information 1.

Supplementary Video 1.

## Data Availability

The original data in this study are available upon request from the corresponding author. The datasets of gene expression data analyzed during the current study are available in the GEO repository, GSE205390.
