# Supplementary material for: IκBζ regulates the development of nonalcoholic fatty liver disease through the attenuation of hepatic steatosis in mice
Source: Sci Rep. 2022 Jul 8;12:11634. doi: 10.1038/s41598-022-15840-0 (PMC9270369; doi:10.1038/s41598-022-15840-0)
Supplement: Supplementary file 1 — Supplementary Information 1. [file 41598_2022_15840_MOESM1_ESM.pdf]

Supplemental Materials

IκBζ Regulates the Development of Nonalcoholic Fatty Liver Disease Through the Attenuation of Hepatic Steatosis in Mice

Hideki Ishikawa, Morisada Hayakawa, Nemekhbayar Baatartsogt, Nao Kakizawa, Hiromi Ohto-Ozaki, Takashi Maruyama, Kouichi Miura, Koichi Suzuki, Toshiki Rikiyama, and Tsukasa Ohmori

Address correspondence and reprint requests to: Tsukasa Ohmori, M.D., Ph.D., Department of Biochemistry, Jichi Medical University School of Medicine; 3311-1 Yakushiji, Shimotsuke, Tochigi 329-0498, Japan. Email: tohmori@jichi.ac.jp. Tel: +81-285-58-7324.

Supplemental Tables 1–4
Supplemental Figures 1–10
Supplemental Video 1：Apoptotic cells in the liver of the mouse model of NAFLD.
Nfkbiz$^{fl/fl}$ and *Alb-Cre; Nfkbiz*$^{fl/fl}$ mice fed with a CDAHFD (High Fat Diet) or an NCD (Normal Diet) for 8 weeks. DAPI and TUNEL staining of the liver sections were observed using a confocal microscope.

Supplemental Table 1. Oligonucleotide primer pairs used in this study

Real time qPCR	For TaqMan Gene Expression Assays with THUNDERBIRD Probe qPCR	
Target Gene		Assay No.
Hprt1		Mm00446968_m1
Nfkbiz		Mm00600522_m1
**Real time qPCR**	**For THUNDERBIRD SYBR qPCR**	
Target Gene		Sequence
Bcl-xl	F	5'- GCTGGGACACTTTTGTGGAT -3'
	R	5'- TGTCTGGTCACTTCCGACTG -3'
Bcl-2	F	5'- TGGGATGCCTTTGTGGAACT-3'
	R	5'- GAGACAGCCAGGAGAAATCA-3'
Bak	F	5'- AGGTGACAAGTGACGGTGGT-3'
	R	5'- AAGATGCTGTTGGGTTCCAG-3'
Bax	F	5'- ACATGTTTGCTGATGGCAAC-3'
	R	5'- CTTCTTCCAGATGGTGAGC-3'
Tgfb1	F	5'- TTTGGAGCCTGGACACACAGTACA -3'
	R	5'- TGTGTTGGTTGTAGAGGGCAAGGA-3'
Pdgfb	F	5'- TGCTGAGCGACCACTCCATC -3'
	R	5'- CTCGGGTCATGTTCAAGTCCA -3'
Timp1	F	5'- GGTGTGCACAGTGTTTCCCTGTTT -3'
	R	5'- TCCCGTCCACAAACAGTGAGTGTCA -3'
Col1a1	F	5'- ACAGTCGCTTCACCTACAGC -3'
	R	5'- TTTTGTATTCGATGACTGTCTTGCC -3'
Acta1	F	5'- GACCGCATGCAGAAGGAGAT -3'
	R	5'- CACACTGAGTACTTGCGCTC -3'
Hprt1	F	5'- GTTGGATACAGGCCAGACTTTGTTG -3'
	R	5'- GATTCAACTTGCGCTCATCTTAGGC -3'
Gapdh	F	5'- CGACTTCAACAGCAACTCCCACTCTTCC -3'
	R	5'- TGGGTGGTCCAGGGTTTCTTACTCCTT -3'
Srebf1		Primer set ID: MA175957 (Perfect Real Time, Takara Bio)
Srebf2		Primer set ID: MA098542 (Perfect Real Time, Takara Bio)
Ppargc1a		Primer set ID: MA197916 (Perfect Real Time, Takara Bio)
Agpat1		Primer set ID: MA203806 (Perfect Real Time, Takara Bio)
Agpat3		Primer set ID: MA226283 (Perfect Real Time, Takara Bio)
Lpin1		Primer set ID: MA224542 (Perfect Real Time, Takara Bio)
Lpin2		Primer set ID: MA216755 (Perfect Real Time, Takara Bio)
Fasn		Primer set ID: MA207469 (Perfect Real Time, Takara Bio)

Human NFKBIZ	F	5'- ATCCAATGAACACCACACAGTTAG -3'
	R	5'- TTGGGCAACAGCAATATGAAG -3'
Human GAPDH	F	5'- GCACCGTCAAGGCTGAGAAC -3'
	R	5'- TGGTGAAGACGCCAGTGGA -3'
Genotyping		
Target Gene		Sequence
Nfkbiz	F1	5'- GCAAATATTCCCCAGGACCAG -3'
	R	5'- GTCTTCACAGCAGGTTATTCACG -3'
	F3	5'- TCAGTGCCAGGTGCGTCTGAG -3'

Supplemental Table 2. Transcripts upregulated by the overexpression of IκBζ in the liver (n = 77)

Gene	Fold change	*P* value
Npm2; Xpo7	24.56	0.0004
Spag17	22.68	0.0086
Trmt2b	22.23	1.56E-05
Gnaz	21.67	9.98E-06
Gm26919	20.16	3.09E-05
Apc2	20.11	0.0057
Lrp2bp	19.28	0.0068
Nfkbiz	18.81	5.39E-05
Prl7d1	18.35	0.0042
Morf4l1; Morf4l1b	17.44	0.0051
Tmem196	17.16	0.007
Gje1	15.03	0.0014
Itk	14.4	0.0056
Hmmr	13.94	0.0003
Obsl1	13.53	0.0092
Ebf1	13.43	0.0058
Msx2	13.3	0.0086
Fgf4	13.25	0.0024
Slc1a3	11.73	0.0086
Ccdc184	10.96	0.0006
Npy5r	10.95	0.0047
Syce1l	10.13	0.0009
Hrnr	10.02	1.00E-05
Myh6; Myh7	9.84	0.0005
Svopl	9.8	0.0015
Nrg2	9.45	0.0003
Fbxl13	9.42	0.0032
Mbd4	9.33	0.0082
Crh	8.81	0.0003
Ccdc18	8.73	0.0066

Pitpnm3	8.34	0.0009
Olfr560; Olfr78	8.34	0.0039
Ccnb3	8.19	0.0052
Lhx1	7.9	0.0015
Fgf2	7.78	0.0008
Cnga1	7.57	0.007
Slc9a8	7.55	0.0009
Cyp2c65	7.55	0.0004
Cyfip2	7.48	0.0009
Wnt9b	7.4	6.32E-05
Tceal7	7.31	0.0014
Dusp18	7.24	0.0003
Fem1a	6.84	0.0076
Dmc1	6.83	0.0007
Eya4	6.76	0.0042
Ceacam18	6.73	3.24E-05
Slc35f4	6.61	0.0058
Col4a3	6.49	0.0048
Cacng8	6.37	0.0044
Brdt	6.34	0.0038
Mak	6.28	0.0029
Svs2	6.27	0.0009
Egln3	6.22	2.61E-05
Dennd6b	6.22	0.0056
Syce1	6.16	0.0055
Ccdc167	6.15	0.005
Utf1	6.15	0.0027
Hsd3b5	6.1	0.004
Prrx1	6.05	0.0009
Gm32930	5.96	0.006
Nck2	5.94	0.002
Ccl12; LOC105242482	5.88	0.0096
Tunar	5.87	0.0084

Mpp4	5.83	0.0038
Hsf2bp	5.72	0.0052
Filip1	5.72	0.0028
Nfat5	5.68	0.0007
Oc90	5.54	0.0038
Ajap1	5.53	7.37E-05
Ly6g6e	5.48	0.007
Zfp248	5.42	0.0013
Adamts4	5.29	0.0036
Eri3	5.29	0.0004
Gadd45gip1	5.16	0.01
Sema6d	5.1	0.0002
Vmn2r88	5.07	0.0009
Krtap9-3	5.06	0.0036

Supplemental Table 3. Transcripts downregulated by the overexpression of IκBζ in the liver (n = 57)

Gene	Fold change	*P* value
Lpin1	−5.05	0.0005
Hs3st3a1	−5.06	0.0047
Arhgef25	−5.13	0.0009
Fbxl18	−5.2	0.0081
Homer1	−5.26	0.0094
Kdm7a	−5.3	0.0081
4933400A11Rik	−5.32	0.0018
Lpin1	−5.32	0.0005
Insm1	−5.42	0.0022
Cldn23	−5.64	0.0099
Gsdma	−5.7	0.0021
Zbtb16	−5.82	0.0001
Slco1a5	−5.85	0.0077
Mark2	−5.98	0.0015
Asb6	−6.03	0.0013
Macc1	−6.07	0.0013
Anks1b	−6.17	0.0094
Plcxd1	−6.23	0.0006
Psma5	−6.3	0.0003
Sncg	−6.35	0.0026
Serpinb12	−6.37	0.009
Pappa	−6.41	0.0022
Clca2	−6.68	0.0094
Tmem132c	−6.82	0.0041
Foxo3	−6.97	3.92E−05
Cts8	−6.99	0.0091
Cr1l	−7.17	0.0052
Apopt1	−7.18	0.0003
Ciart	−7.2	0.0038
Gm38718; Gm39869; Ptprj	−7.55	0.0017

Gpr135	−7.95	0.0006
Eef2	−7.97	0.0001
Actl9	−8.26	0.0062
Tmod2	−8.45	0.0001
Eml6	−8.52	9.22E−05
Rnf169	−8.56	0.0054
Fastkd5; Ubox5	−8.67	0.0019
Zbtb16	−8.8	4.31E−05
Tssk6	−8.87	0.0079
Dbx1	−9.21	0.0016
Brdt	−9.45	0.004
Plpp4	−9.94	0.0002
Trappc12	−10.38	0.0007
Lrp8	−10.73	0.0004
Cend1	−10.89	0.0022
Prtg	−10.94	0.0017
Fkbp5	−11.1	0.0027
Elavl4	−11.22	0.0066
Mmp16	−11.28	0.0019
Grid1	−11.7	0.0008
Nup153	−12.06	9.42E-05
Vldlr	−14.17	2.32E-05
Cdk2	−16.06	0.0023
Adamtsl1	−16.47	0.001
Rere	−16.49	0.0017
Rln1	−17.22	0.0021
Itgb1	−20.75	0.0009

Supplemental Table 4. Changes in transcripts related to β-oxidation by the overexpression of IκBζ in the liver

Gene	Fold change	*P* value
Cpt2	1.64	0.5359
Ehhadh	1.6	0.1733
Hadh	1.41	0.7988
Hadhb	1.24	0.2583
Echs1	1.23	0.4816
Hadhb	1.22	0.3472
Acaa1a	1.21	0.7724
Acaa2	1.2	0.3924
Cpt1a	1.19	0.5622
Chkb; ChkbCpt1b; Cpt1b	1.15	0.4185
Acaa1a; Acaa1b	1.1	0.3946
Acaa1b	1.09	0.5395
Acaa1a; Acaa1b	1.05	0.7725
Acaa2	1.05	0.8936
Acaa1a	1.04	0.7478
Acadvl	1.02	0.9176
Hadh	1.02	0.73
Hadh	1.01	0.6174
Cpt1a	1.01	0.4647
Acaa2	-1.03	0.9216
Hadha	-1.09	0.5858
Cpt2	-1.15	0.749
Cpt1a	-1.17	0.7872
Cpt1c	-2.67	0.4384
Hadhb	-5.73	0.2778

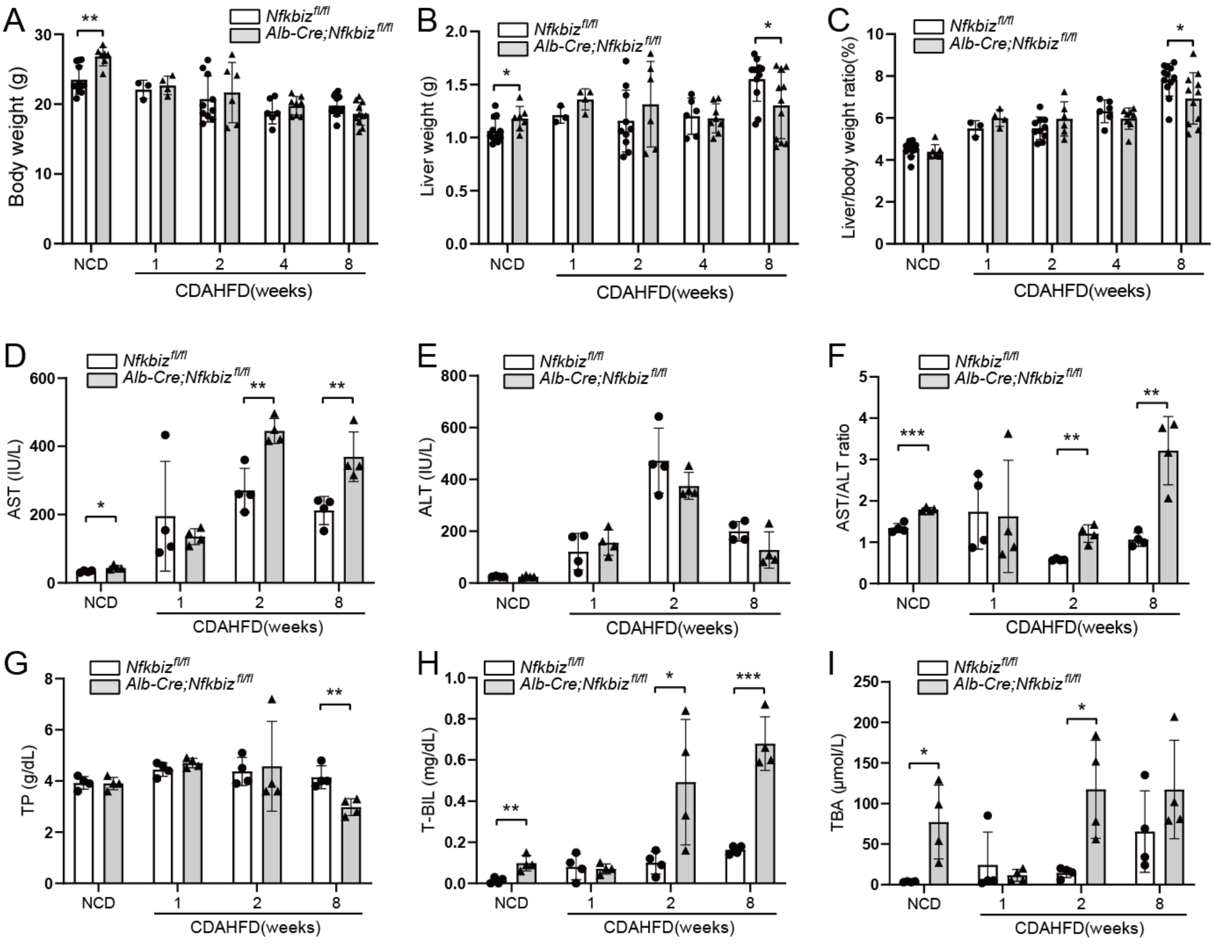

Supplemental Figure 1 Deficiency of IκBζ in hepatocyte-aggravated plasma clinical chemistry parameters in a mouse model of NAFLD. *Nfkbiz*$^{fl/fl}$ (white bar) and *Alb-Cre; Nfkbiz*$^{fl/fl}$ (black bar) mice were fed a CDAHFD for 8 weeks. (**A, B, C**) Changes in body weight (**A**), liver weight (**B**), and the relative ratio of liver weight to body weight (**C**). Values are expressed as the mean ± SD (n = 3–12 for each point). (**D, E, F, G, H, I)** Blood was drawn from mice at 0, 1, 2, and 8 weeks after the CDAHFD challenge. Plasma aspartate aminotransferase (AST) (**D**), alanine aminotransferase (ALT) (**E**), AST/ALT ratio (**F**), total protein (TP) (**G**), total bilirubin (T-BIL) **(H)**, and total bile acids (TBA) (**I**) were measured. Values are expressed as the mean ± SD (n = 4). *P < 0.05, **P < 0.01, ***P < 0.001. CDAHFD, choline-deficient, L-amino acid-defined, high-fat diet; NCD, normal chow diet.

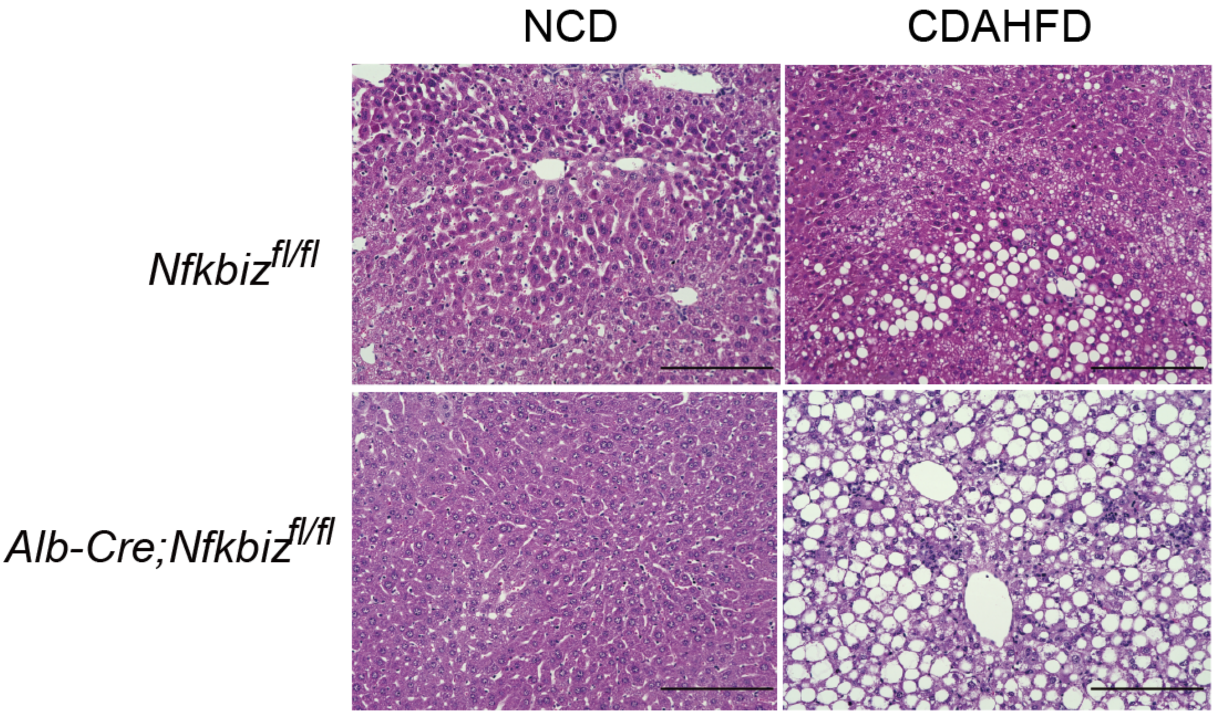

Supplemental Figure 2 *Alb-Cre; Nfkbiz*$^{fl/fl}$ mice have accelerated the development of NAFLD. C57BL/6 mice were fed an NCD or a CDAHFD. Enlarged images of the liver (H&E staining) of Fig. 1E obtained from *Nfkbiz*$^{fl/fl}$ and *Alb-Cre; Nfkbiz*$^{fl/fl}$ mice fed a CDAHFD or an NCD for 2 weeks. Bar = 200 µm. CDAHFD, choline-deficient, L-amino acid-defined, high-fat diet; NCD, normal chow diet.

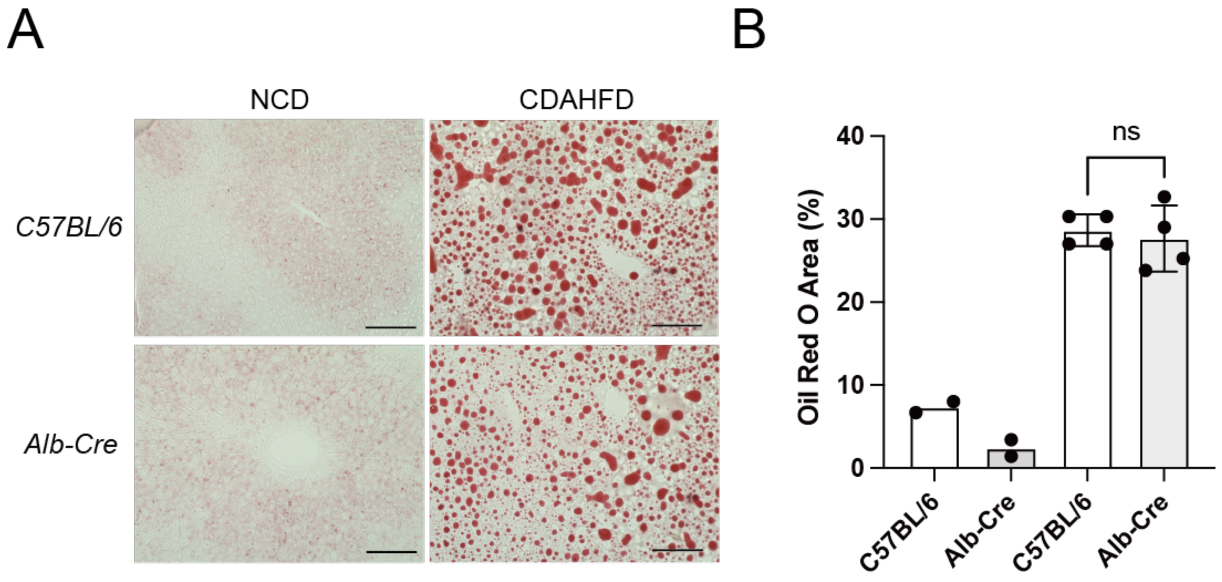

Supplemental Figure 3 Cre expression alone in the liver does not enhance liver steatosis. C57BL/6 and *Alb-Cre* mice were fed an NCD or CDAHFD for 2 weeks. **(A)** Representative image of Oil Red O staining of liver sections from mice fed CDAHFD or NCD for 2 weeks. Bar = 100 µm. **(B)** Bar graph of the Oil Red O staining areas quantified using BZ-X 700 imaging software (Keyence). The Oil red O staining areas were expressed as a percentage of the total area. Values are expressed as the mean ± SD (NCD, n = 2; CDAHFD, n = 4). CDAHFD, choline-deficient, L-amino acid-defined, high-fat diet; NCD, normal chow diet.

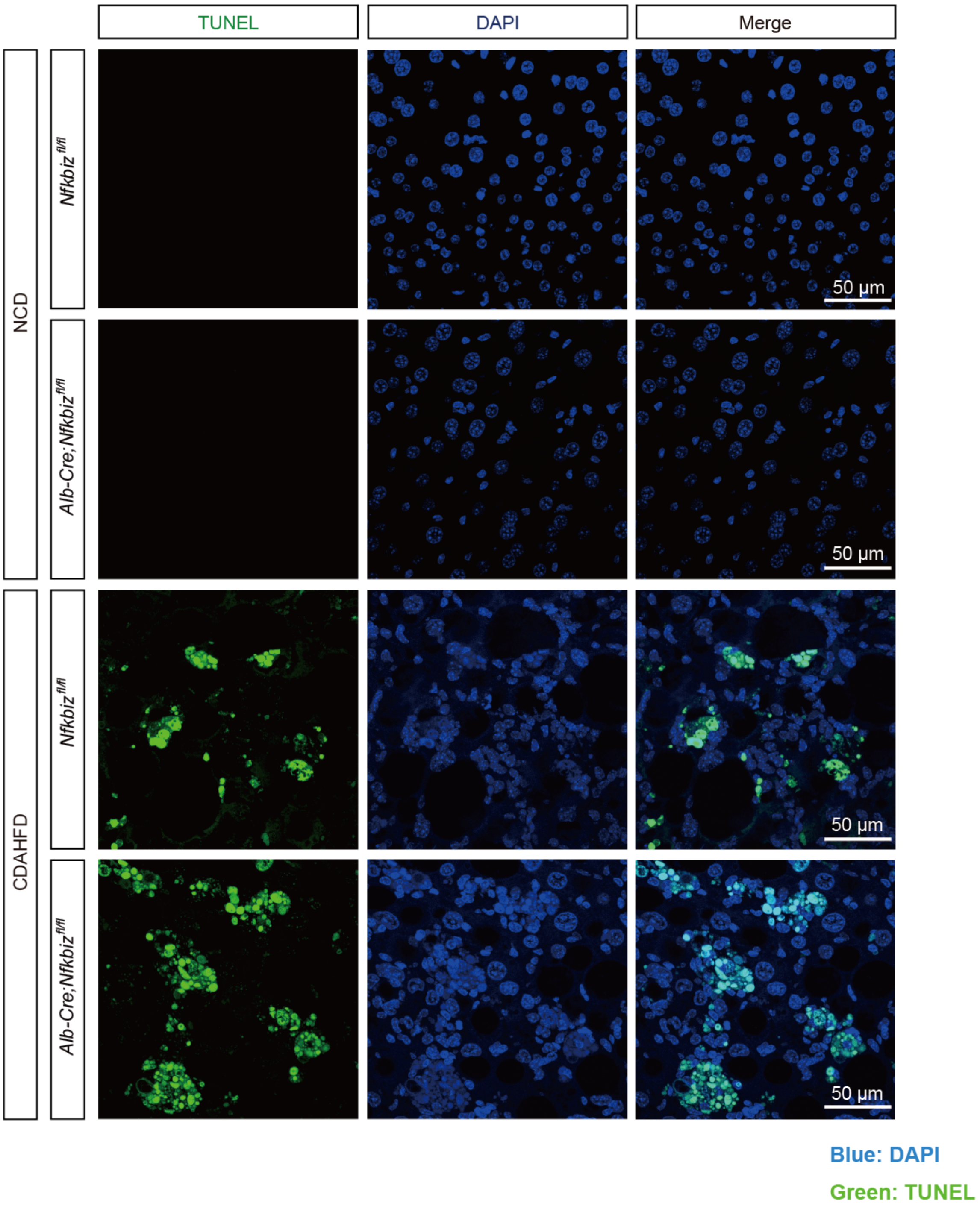

Supplemental Figure 4 The deficiency of IκBζ in hepatocytes increases apoptotic cells in the liver of the mouse model of NAFLD. *Nfkbiz*$^{fl/fl}$ and *Alb-Cre; Nfkbiz*$^{fl/fl}$ mice were fed a CDAHFD or an NCD for 8 weeks. TUNEL staining of the liver section was observed using a confocal microscope (Leica TCS SP8; Leica Microsystems). Representative image of TUNEL staining of liver sections from mice fed a CDAHFD or an NCD for 8 weeks. Green, apoptotic cells; Blue, DAPI. Bar = 50 µm. CDAHFD, choline-deficient, L-amino acid-defined, high-fat diet; NCD, normal chow diet.

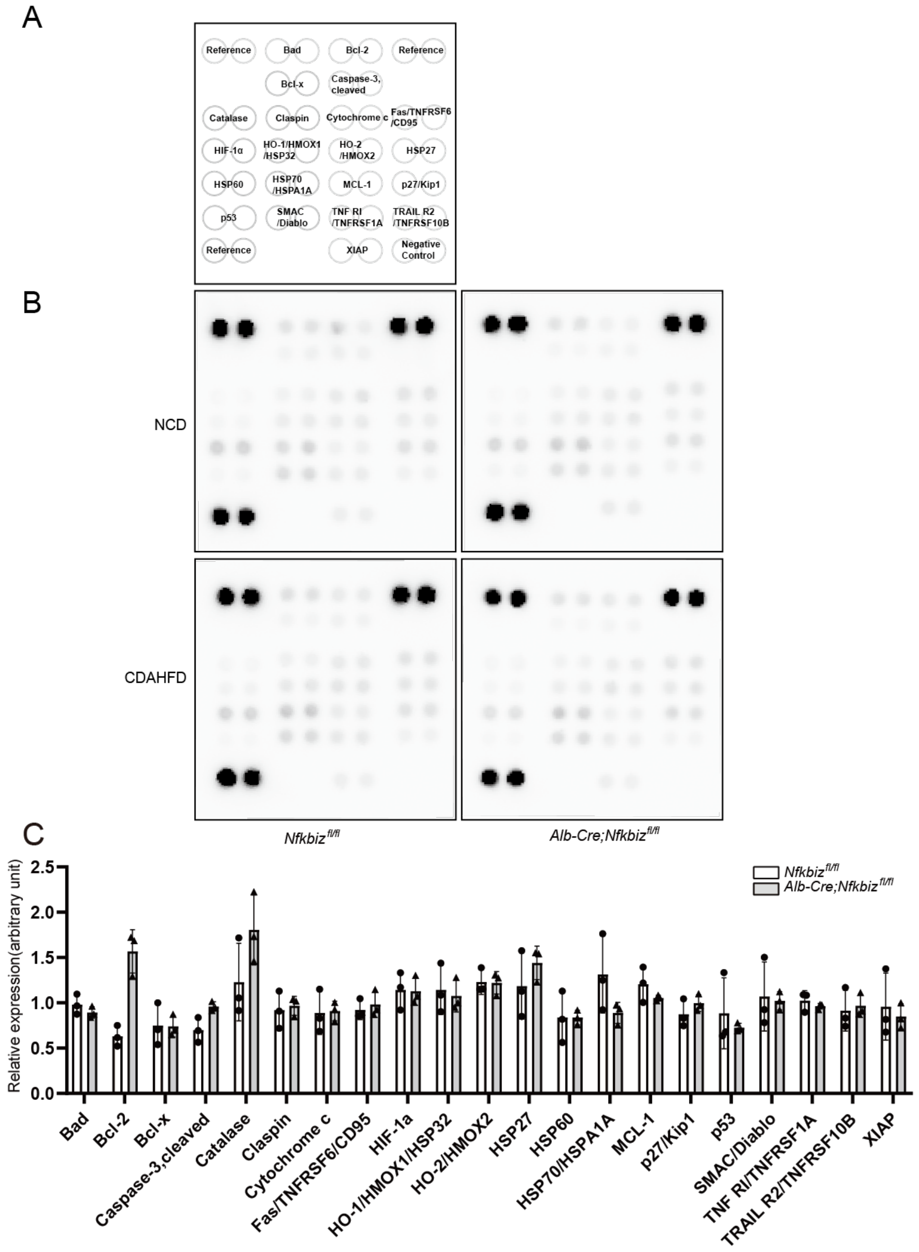

Supplemental Figure 5 The expression of apoptosis-related proteins in the liver. The expression of apoptosis-related proteins in the liver of mice fed CDAHFD or NCD for 4 weeks was assessed by a Proteome Profiler mouse apoptosis Array Kit (R&D Systems). (**A**) The location of the spots for each protein in the array membrane is shown. (**B**) Representative blotting obtained from the liver of *Nfkbiz*$^{fl/fl}$ mice (Left) or *Alb-Cre; Nfkbiz*$^{fl/fl}$ (Right) treated with NCD (Upper panel) or CDAHFD for 4 weeks (Lower Panel). **(C)** The relative expression of each cytokine at 4 weeks after the CDAHFD challenge was quantified from pixel densities using an ImageQuant LAS4000 system (GE Healthcare) (white bar, *Nfkbiz*$^{fl/fl}$; gray bar, *Alb-Cre; Nfkbiz*$^{fl/fl}$). Values are expressed as the mean ± SD (n = 3). CDAHFD, choline-deficient, L-amino acid-defined, high-fat diet; NCD, normal chow diet.

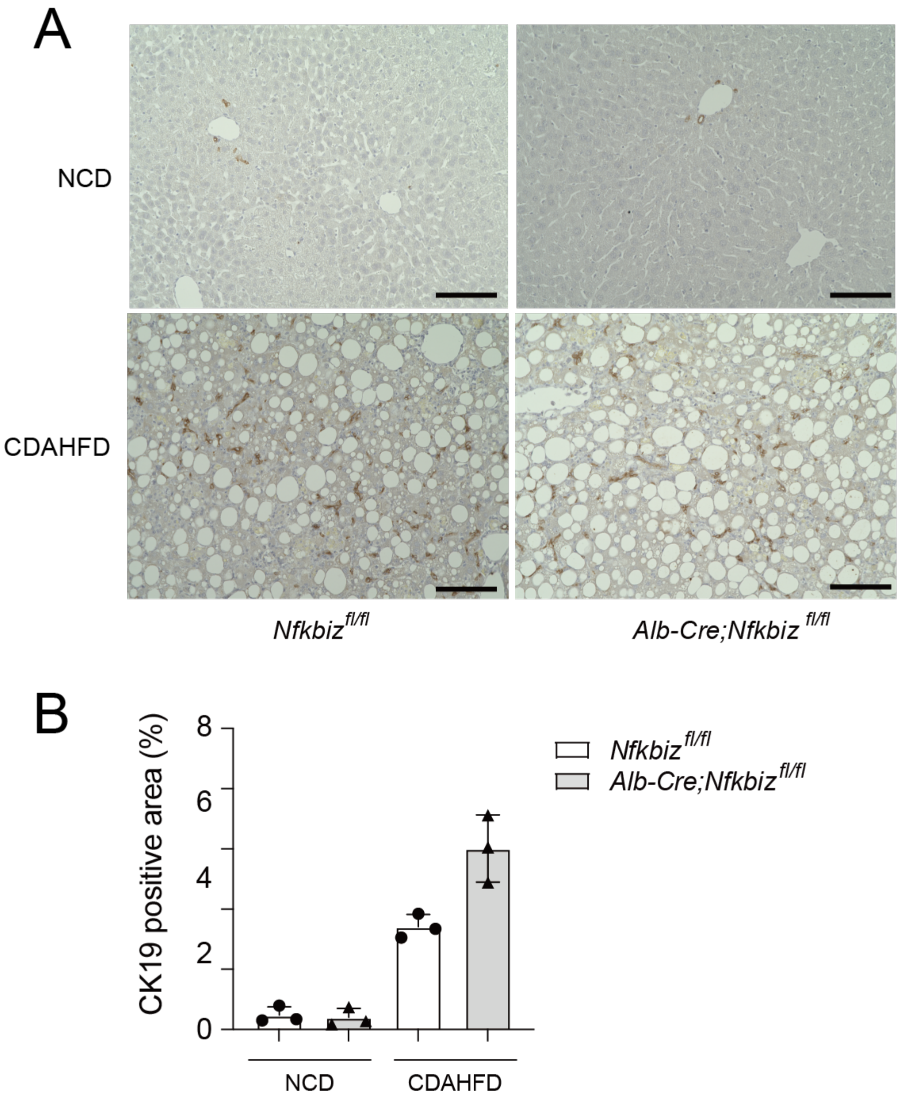

Supplemental Figure 6 The increase in CK19-positive cells in the mouse model of NAFLD. *Nfkbiz*$^{fl/fl}$ and *Alb-Cre; Nfkbiz*$^{fl/fl}$ mice were fed CDAHFD or NCD for 8 weeks. CK19 expression in the liver was assessed by immunohistochemical analysis. Sections were observed using an all-in-one microscope (BZ-X 700, Keyence). (**A**) Representative image of CK19 staining of liver sections from mice fed CDAHFD or NCD for 8 weeks. Braun, CK-19; Blue, Mayer's hematoxylin. Bar = 100 µm. **(B)** CK19-positive area quantified using BZ-X 700 imaging software (Keyence). Values are expressed as the mean ± SD (n = 3). CDAHFD, choline-deficient, L-amino acid-defined, high-fat diet; NCD, normal chow diet.

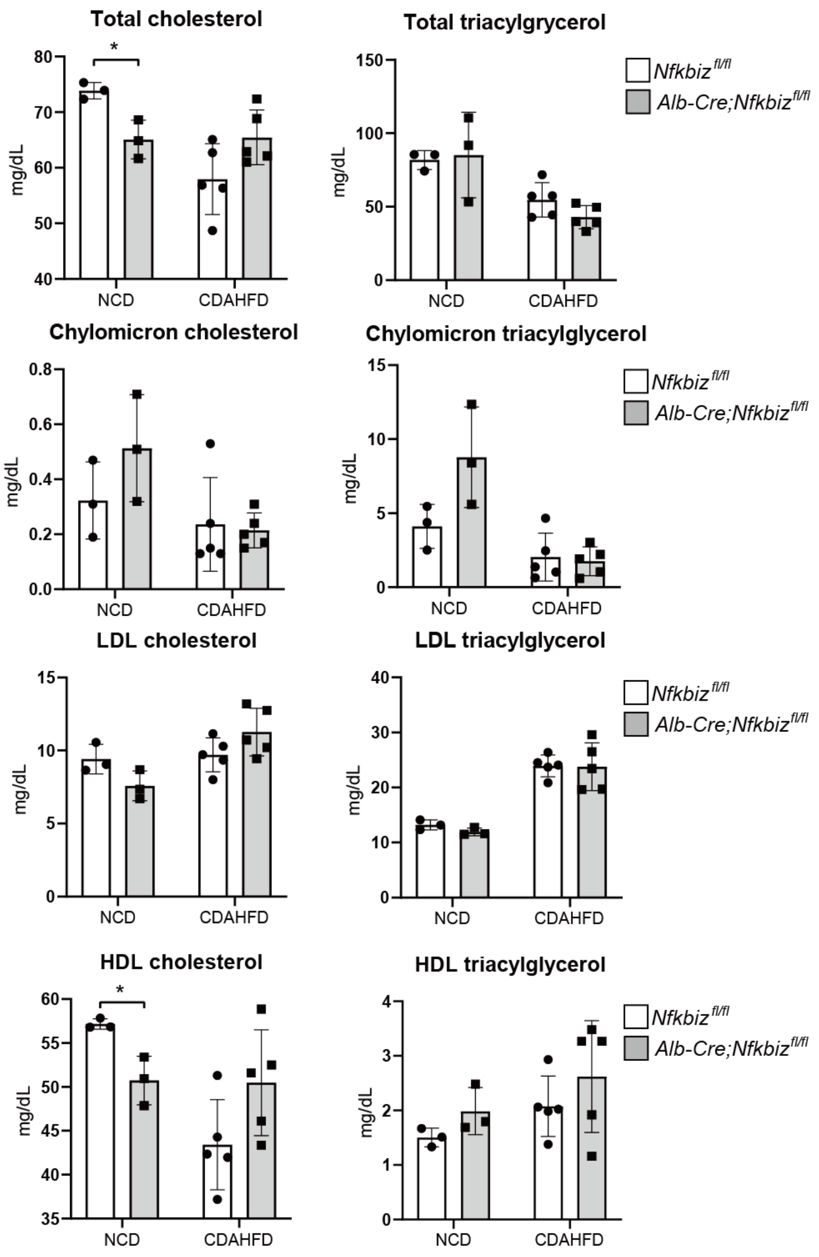

Supplemental Figure 7 Plasma concentrations of each lipoprotein fraction. *Nfkbiz*$^{fl/fl}$ and *Alb-Cre; Nfkbiz*$^{fl/fl}$ mice were fed CDAHFD or NCD for 2 weeks. Plasma lipoprotein fractions were analyzed by gel high-performance liquid chromatography. Values are mean ± SD (n = 3–5). *$P < 0.05$. CM, chylomicron; VLDL, very low-density lipoprotein; LDL, low-density lipoprotein; HDL, high-density lipoprotein. CDAHFD, choline-deficient, L-amino acid-defined, high-fat diet; NCD, normal chow diet.

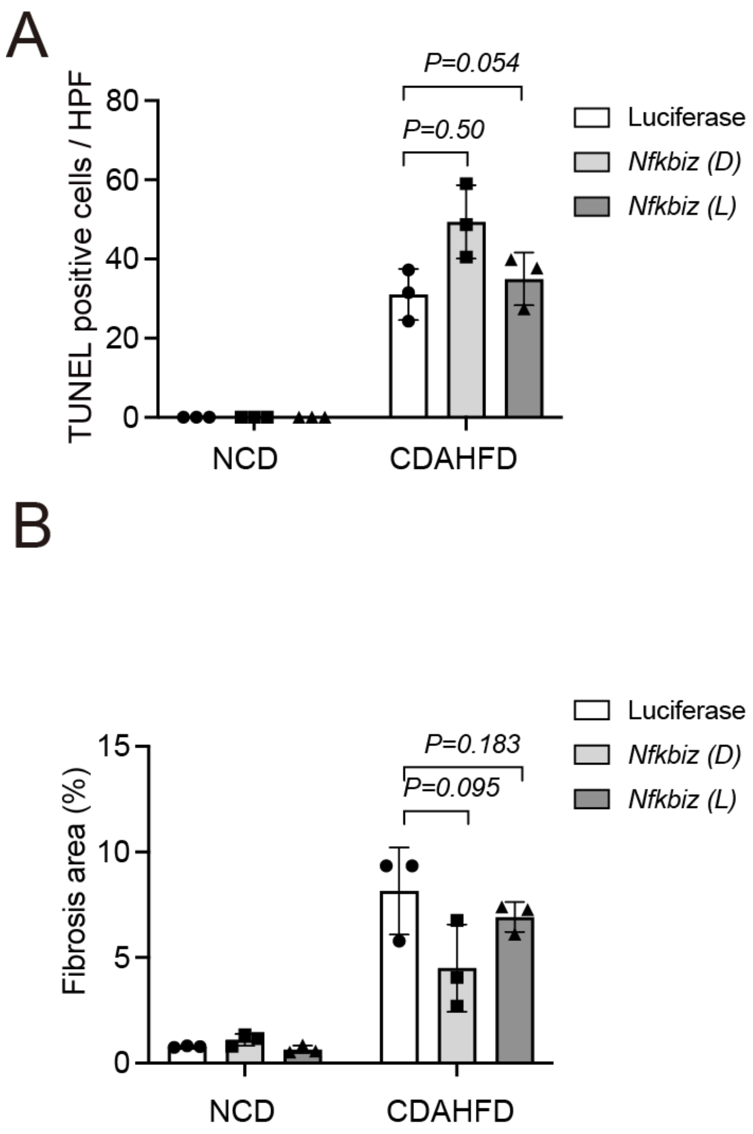

Supplemental Figure 8 Overexpression of *Nfkbiz* in liver hepatocytes by AAV8 vector does not ameliorate hepatic apoptosis and fibrosis *in vivo*. C57BL/6 mice were treated with 3×10^{11} vg of AAV8 vector harboring *Luciferase*, *Nfkbiz* (D), or *Nfkbiz* (L), and then challenged with CDAHFD or NCD for 4 weeks. **(A)** The number of apoptotic cells was assessed by TUNEL staining in one section at high magnification from mice fed CDAHFD or NCD. Values are expressed as the mean ± SD (n = 3). **(B)** Sirius red-positive area was quantified, and the fibrosis area was expressed as the percentage of the Sirius red-positive area. Values are expressed as the mean ± SD (n = 3). *Nfkbiz (D)*, deletion mutant of IκBζ lacking the trans-activating domain; *Nfkbiz (L)*, full-length IκBζ; CDAHFD, choline-deficient, L-amino acid-defined, high-fat diet; NCD, normal chow diet.

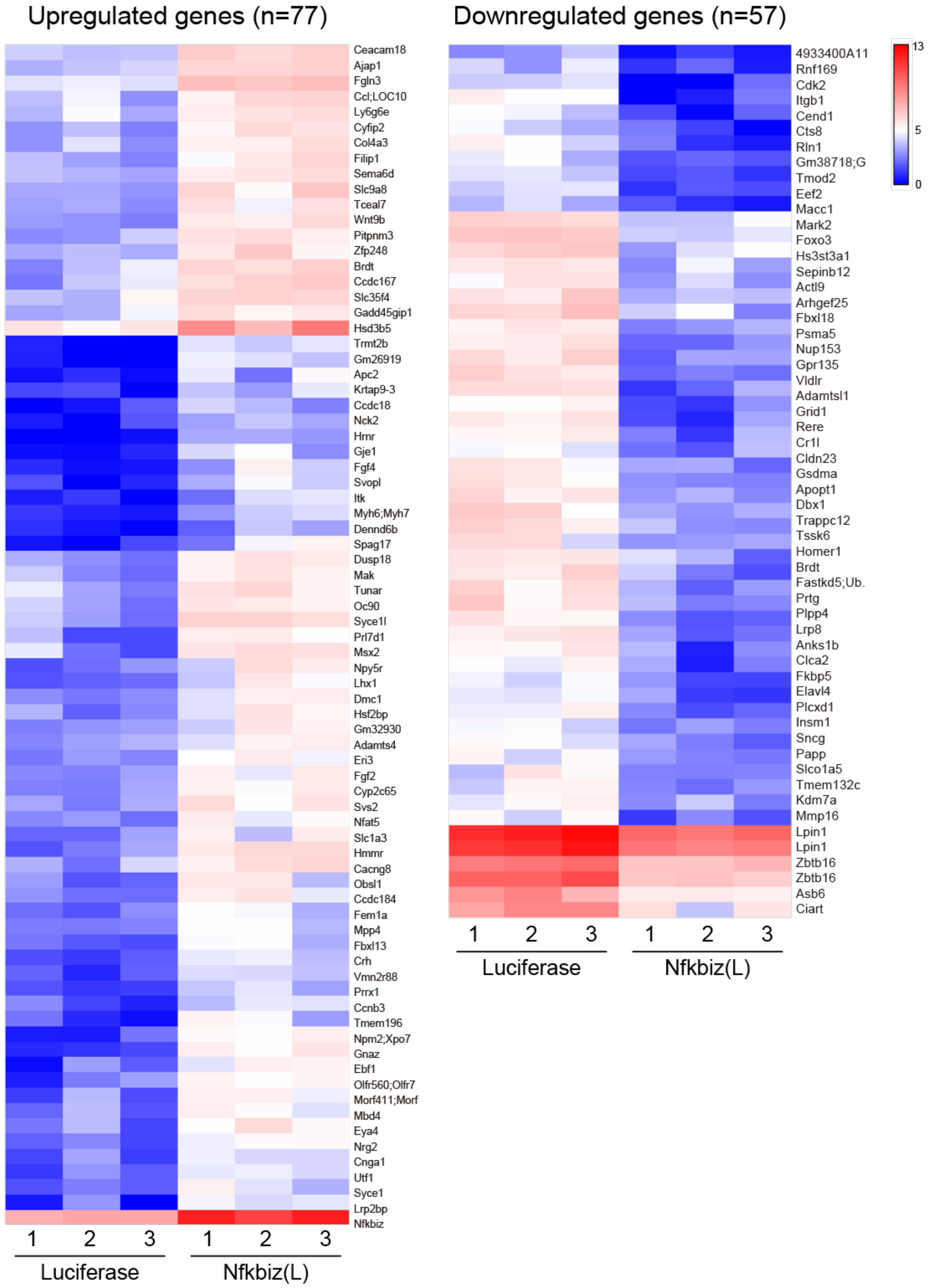

Supplemental Figure 9 Microarray analysis in the liver of mice treated with AAV8 vector harboring *Nfkbiz*. C57BL/6 mice (7 weeks old) were intravenously treated with 3×10^{11} vg of AAV8 vector harboring *Luciferase* or *Nfkbiz* (L). The expression of transcripts in the liver at 4 weeks after vector injection was analyzed by Mouse Genome 430 2.0 Array (n = 3 in each group). The hierarchical cluster analysis of statistically upregulated (left) and downregulated genes (right) were shown (>5 fold, $P < 0.01$).

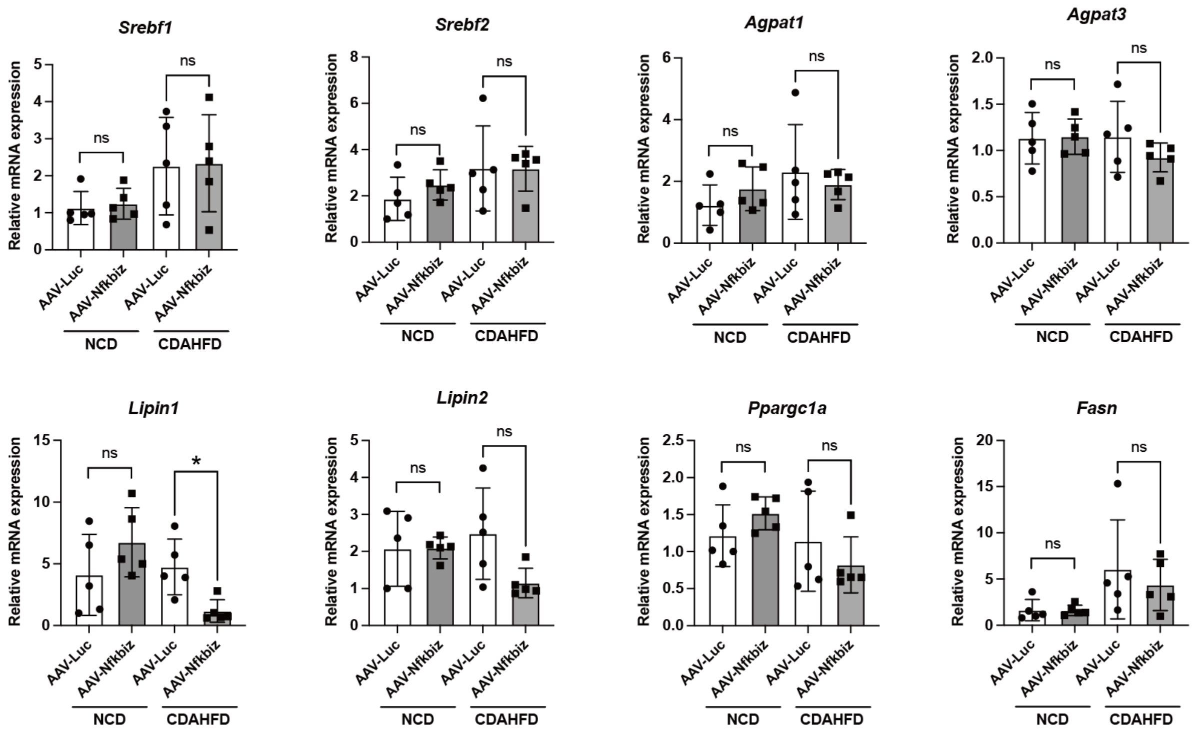

Supplemental Figure 10 Quantitative RT-PCR of mRNA selected from the microarray analysis in the liver of mice treated with the AAV8 vector harboring *Nfkbiz*. C57BL/6 mice (7 weeks old) received an intravenous injection of 3×10^{11} vg of AAV8 vector harboring *Luciferase* (AAV-Luc) or *Nfkbiz* (L) (AAV-Nfkbiz). The NCD or CDAHFD challenge started at 4 weeks after the vector injection. Liver RNA was isolated from mice at 4 weeks after the challenge. The mRNA of *Srebf1*, *Srebf1*, *Agpat1*, *Agpat3*, *Lipin1*, *Lipin2*, *Ppargc1a*, and *Fasn* was analyzed by quantitative RT-PCR and was expressed as the fold increase in the *Nfkbiz*/*Hprt1* ratio. Values are expressed as the mean ± SD (n = 5). $^*P < 0.05$. CDAHFD, choline-deficient, L-amino acid-defined, high-fat diet; NCD, normal chow diet.
